# The GTP- and Phospholipid-Binding Protein TTD14 Regulates Trafficking of the TRPL Ion Channel in *Drosophila* Photoreceptor Cells

**DOI:** 10.1371/journal.pgen.1005578

**Published:** 2015-10-28

**Authors:** Alexander C. Cerny, André Altendorfer, Krystina Schopf, Karla Baltner, Nathalie Maag, Elisabeth Sehn, Uwe Wolfrum, Armin Huber

**Affiliations:** 1 Department of Biosensorics, Institute of Physiology, University of Hohenheim, Stuttgart, Germany; 2 Department of Cell and Matrix Biology, Institute of Zoology, Johannes Gutenberg-University of Mainz, Mainz, Germany; New York University, UNITED STATES

## Abstract

Recycling of signaling proteins is a common phenomenon in diverse signaling pathways. In photoreceptors of *Drosophila*, light absorption by rhodopsin triggers a phospholipase Cβ-mediated opening of the ion channels transient receptor potential (TRP) and TRP-like (TRPL) and generates the visual response. The signaling proteins are located in a plasma membrane compartment called rhabdomere. The major rhodopsin (Rh1) and TRP are predominantly localized in the rhabdomere in light and darkness. In contrast, TRPL translocates between the rhabdomeral plasma membrane in the dark and a storage compartment in the cell body in the light, from where it can be recycled to the plasma membrane upon subsequent dark adaptation. Here, we identified the gene mutated in *trpl translocation defective 14* (*ttd14*), which is required for both TRPL internalization from the rhabdomere in the light and recycling of TRPL back to the rhabdomere in the dark. TTD14 is highly conserved in invertebrates and binds GTP *in vitro*. The *ttd14* mutation alters a conserved proline residue (P75L) in the GTP-binding domain and abolishes binding to GTP. This indicates that GTP binding is essential for TTD14 function. TTD14 is a cytosolic protein and binds to PtdIns(3)P, a lipid enriched in early endosome membranes, and to phosphatidic acid. In contrast to TRPL, rhabdomeral localization of the membrane proteins Rh1 and TRP is not affected in the *ttd14* ^*P75L*^ mutant. The *ttd14* ^*P75L*^ mutation results in Rh1-independent photoreceptor degeneration and larval lethality suggesting that other processes are also affected by the *ttd14* ^*P75L*^ mutation. In conclusion, TTD14 is a novel regulator of TRPL trafficking, involved in internalization and subsequent sorting of TRPL into the recycling pathway that enables this ion channel to return to the plasma membrane.

## Author Summary

Protein trafficking in neurons occurs throughout the lifetime of a cell and includes the internalization and redistribution of plasma membrane proteins. Regulated protein trafficking controls the equipment of the plasma membrane with receptors and ion channels and thereby attenuates or enhances neuronal function. Defects in recycling of plasma membrane proteins can cause detrimental neurodegenerative diseases such as Alzheimer’s disease, Parkinson’s disease and Down´s syndrome. In *Drosophila* photoreceptors, the TRPL ion channel, together with the TRP channel, mediates vision and light-dependently shuttles between an endomembrane storage compartment and the apical plasma membrane. Here, we report the identification of a mutation in the *ttd14* gene that inhibits TRPL-trafficking in both directions and also results in photoreceptor degeneration. The TTD14 protein contains a region with weak homology to a PX-domain, which is also found in proteins that sort cargo in the endosome and enable protein recycling. We characterize TTD14 as a new regulator of photoreceptor maintenance and ion channel trafficking that binds to GTP and PtdIns(3)P, a phospholipid enriched in early endosomes.

## Introduction

Photoreceptor membrane proteins undergo a carefully regulated turnover that helps to adjust the sensitivity of the receptors and to renew old and possibly worn out proteins. Throughout the lifetime of a photoreceptor cell, new proteins are synthesized and transported to the photoreceptive membrane while other proteins are removed from this membrane and are either recycled or degraded in the lysosome. Major integral membrane proteins of the *Drosophila* photoreceptive membrane comprise the G protein-coupled receptor rhodopsin and two ion channels, transient receptor potential (TRP) and TRP-like (TRPL). Defects in rhodopsin turnover can result in degeneration of photoreceptors in humans and flies [1–3]. The signaling cascade operating in fly photoreceptors is a G protein-coupled, phospholipase Cβ-mediated signaling pathway that is initiated by the absorption of a photon by rhodopsin and results in the opening of TRP and TRPL channels and subsequent influx of sodium and calcium ions. TRP and TRPL are the founding members of the large family of transient receptor potential channels that comprises 28 members in mammals [4–7]. TRP channels function in sensory systems as well as in calcium regulation in non-neuronal cells, for example in kidney or heart cells.

In *Drosophila*, newly synthesized rhodopsin of R1-6 photoreceptor cells (Rh1) and the two light-activated ion channels TRP and TRPL are transported via the secretory pathway from the endoplasmic reticulum (ER) to the apical plasma membrane that forms a light-sensitive microvillar compartment, termed rhabdomere. Precise folding and successful transport of Rh1 and TRP channels to the rhabdomere are crucial for photoreceptor function. A number of reports have identified proteins required for the anterograde transport of Rh1 and for its endocytosis [8–19]. These include chaperones, Rab GTPases (Rab1, Rab6, Rab11), Rab-interacting proteins, a COPII-interacting phosphatidic acid phospholipase A1, and myosin V. Among these proteins, the chaperone XPORT, Rab11, and the Rab11-interacting guanine nucleotide exchange factor Crag were shown to also be required for TRP trafficking, but not for TRPL trafficking [9, 15, 18]. Although Rh1 undergoes a turnover that is enhanced in the light most of the Rh1 is detected in the rhabdomeres in both light- and dark-adapted flies. A significant portion of Rh1 that is removed from the rhabdomere in the light ends up in the lysosome and becomes degraded [20–22]. However, the rhabdomeral Rh1 content does not change significantly under physiological light conditions, indicating that degraded Rh1 is replenished by newly synthesized protein.

It has recently been reported that a fraction of internalized Rh1 is not degraded but enters a recycling pathway that requires components of the retromer complex [23]. The retromer is a hetreromultimeric protein complex composed of Vps26, Vps29, Vps35, and sorting nexins [24–27]. It is a principle component of the retrograde transport from endosomes to the trans-Golgi network or for recycling of proteins to the plasma membrane for many recycling membrane proteins, including Wntless [28], the β2 adrenoreceptor [29], the *Drosophila* adherens junction protein Crumbs [30], and vertebrate AMPA receptor subunits [31–33]. Wang et al. [23] showed that Vps26 and Vps35 are required for Rh1 recycling and that mutations in these retromer proteins cause retinal degeneration.

The *Drosophila* ion channel TRPL is a recycling photoreceptor membrane protein that undergoes light-dependent translocation between the rhabdomere, where it is located in dark-adapted flies, and a storage compartment in the cell body, to which it is transported upon illumination within several hours [34]. The removal of TRPL from the rhabdomere depends on activation of the phototransduction cascade and the resulting Ca^2+^ influx through TRP channels [35]. It has been described as a two-step process in which TRPL first (within 5–10 minutes) is transported to the base of the microvilli and adjacent stalk membrane via lateral membrane transport and then (within several hours) becomes internalized by a vesicular transport mechanism [36–38]. The majority of internalized TRPL does not enter the lysosomal pathway but is stored in the cell body and recycled back to the rhabdomere when the flies are transferred from light to darkness [34, 37].

In order to identify components required for TRPL transport, we previously performed a genetic screen for TRPL translocation-defective mutants [39]. This screen used a TRPL-eGFP reporter gene to monitor TRPL localization in intact flies and was based on FRT/FLP-driven mitotic recombination enabling the generation of homozygous mutant eye clones of otherwise lethal genes. In the present study, we mapped the mutant *ttd14* and identified the mutated gene. The encoded protein TTD14 is a cytosolic GTP-binding protein that interacts with phospholipids and functions in the internalization and recycling of TRPL during light-triggered TRPL translocation between cell body and rhabdomere. In addition, the *ttd14* mutant results in photoreceptor degeneration and larval lethality indicating a vital role of *ttd14* in other contexts.

## Results

### The homozygous lethal mutation *ttd14* maps to the gene CG30118 and affects TRPL trafficking.

**Fig 1:**
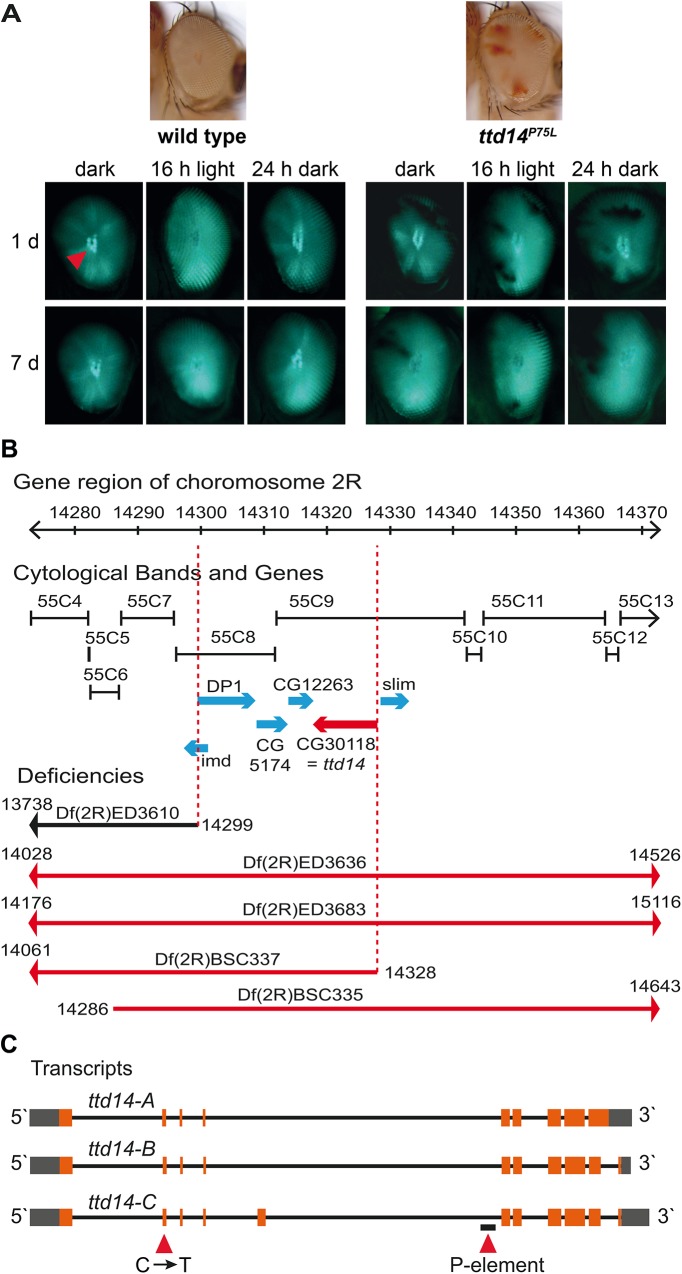
Deficiency mapping of the *ttd14* locus. (A) Images on top show a wild type eye and a mosaic eye with white homocygous *ttd14* ^*P75L*^ cell clones and red heterozygous cell clones. The panels show deep pseudopupils (red arrowhead) as revealed by TRPL-eGFP fluorescence in the eyes of wild type flies and *ttd14* mutant eye clones. Flies were raised in the dark for 1 day (upper row) or 7 days (lower row) and then subjected to 16 hours of orange light. Following orange light adaptation flies were again kept in darkness for 24 hours. (B) Using deletion strains from the Bloomington stock collection the lethality of *ttd14* was mapped to the region 55C8–55C9 on chromosome 2R. The gene region, the corresponding cytological bands, the position of genes in the mapped region as annotated in flybase (http://flybase.org), and the localization of deficiencies that complemented (black) or failed to complement (red) the lethality of *ttd14* are shown. (C) Three different mRNAs are predicted to be transcribed from the *ttd14* gene. In the transcript schemes, orange and gray boxes denote protein coding and non-coding exons, respectively. Black lines represent introns. *ttd14*-A and -B differ at the 3`end encoding proteins of 475 and 471 amino acids, respectively. *ttd14*-C has an additional exon and encodes a protein of 515 amino acids. The C to T mutation at position 751 and a P-element insertion (*CG30118* ^*KGO3769*^) are indicated by red arrowheads.

**Fig 2:**
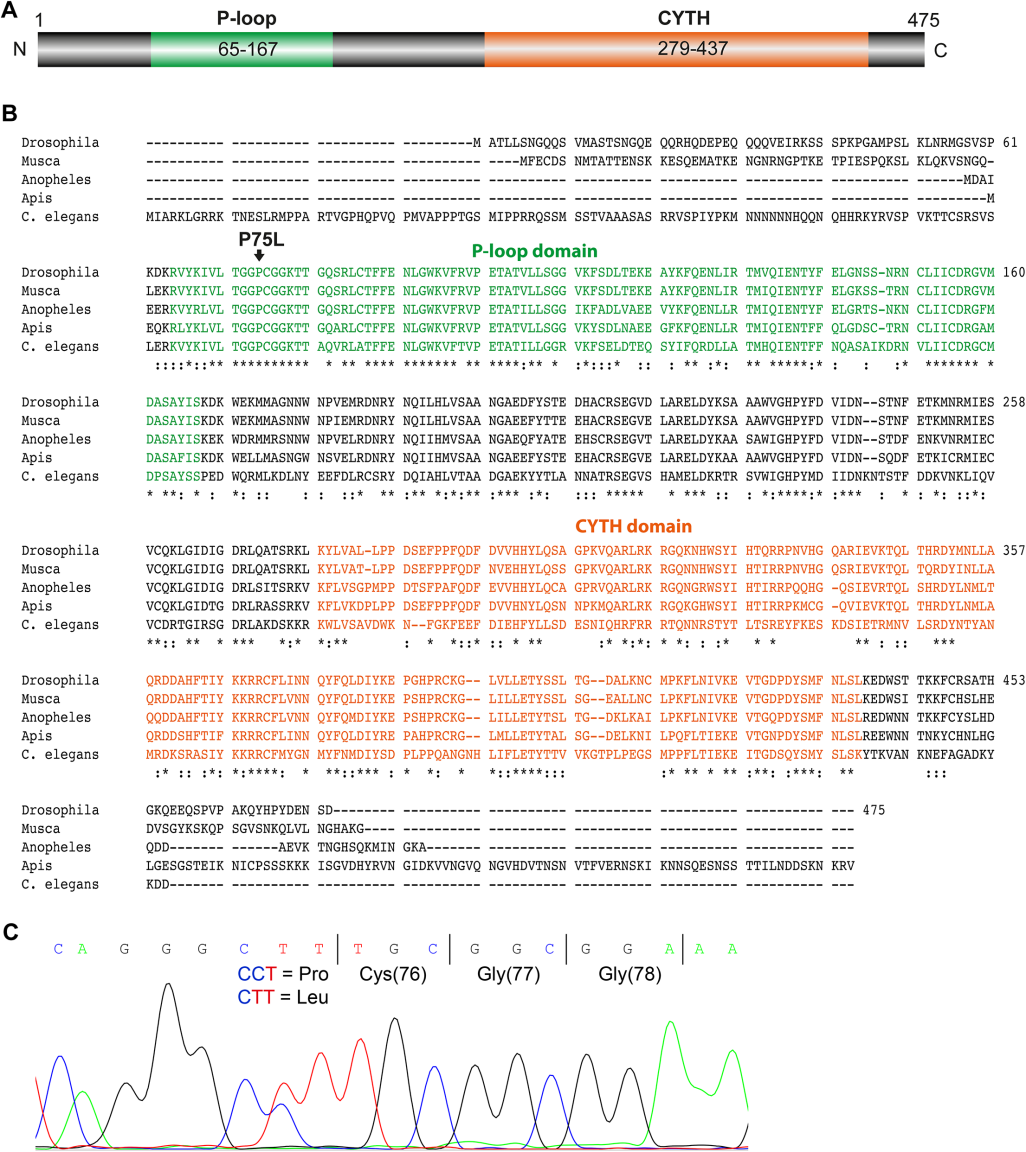
Amino acid sequence analysis of TTD14. (A) Scheme of the 475 amino acid long TTD14-A protein. The scheme illustrates a P-loop nucleoside triphosphate hydrolase domain (P-loop) spanning amino acids 65–167 and a CYTH domain (CYTH) containing amino acids 279–437 as predicted by InterPro (http://www.ebi.ac.uk/interpro). (B) Amino acid sequence alignment of TTD14 homologs from *Drosophila melanogaster* (isoform A), *Musca domestica*, *Anopheles gambiae*, *Apis mellifera*, and *Caenorhabditis elegans*. * denotes amino acids identical in all sequences,: denotes conserved substitutions. The P-loop containing nucleoside triphosphate binding domain (green), a CYTH-like domain (orange), and the P75L point mutation (arrow) are indicated. (C) Electropherogram of a sequencing reaction from genomic DNA of a heterozygous *ttd14* mutant. The wild type and the mutant allele have, respectively, a CCT (encoding proline) or a CTT codon (encoding leucine) at amino acid position 75.

The recessive mutant *ttd14* is homozygous lethal during the larval stage and was obtained from a genetic screen of chromosome arm 2R that used ethyl methanesulfonate as a mutagene and was based on FRT/FLP-driven mitotic recombination of the chromosome arm to obtain homozygous mutant eye clones [39]. In the genetic screen, trafficking of the TRPL-eGFP reporter protein was visualized in mosaic eyes by the fluorescence of the deep pseudopupil and the *ttd14* mutant was identified by its defective TRPL-eGFP internalization to the cell body after 16 hours orange light illumination. A detailed description of the screen, including crossing schemes, can be found in Ref. [39]. A more detailed analysis using age-matched flies revealed a complex phenotype including defective light-induced TRPL internalization to the storage compartment in young flies, TRPL depletion in the rhabdomere after long-term dark adaptation, and defective redistribution of TRPL to the rhabdomere upon light adaption and subsequent dark adaptation (Fig 1A). To identify the gene affected in the *ttd14* mutant, we made use of the lethal phenotype of *ttd14*, assuming that lethality in larvae and impaired TRPL trafficking in mosaic eye clones is caused by the same mutation. We performed a mapping approach of the mutated chromosome arm 2R with deficiency strains. A lethal mutation could be mapped between 55C8–55C9, a region of 30 kilobases containing six candidate genes (Fig 1B). Among these candidate genes, a lethal P-Element mutation in the orphan gene *CG30118* (*CG30118* ^*KG03769*^, see Fig 1C) failed to complement the lethality of the *ttd14* mutant demonstrating that *ttd14* is a mutant allele of the *CG30118* gene. As shown in Fig 1C, the *ttd14* gene encodes three predicted transcripts (*ttd14-*A, *ttd14*-B, and *ttd14*-C). Sequencing of the coding sequence of the *ttd14* gene derived from cDNA of wild type fly heads confirmed expression of *ttd14-*A and -B but not *ttd14*-C in *Drosophila* heads. Sequencing of genomic DNA obtained from a heterozygous *ttd14* mutant revealed that the mutation present in the *ttd14* allele is a C → T transition in the second exon altering the codon for proline75 to a codon for leucine (P75L) (Fig 2C). This mutation affects all *ttd14* transcripts as P75 is encoded by a common exon. In order to demonstrate that the lethal phenotype and the TRPL transport defect are caused by the same mutation in *ttd14*, we generated myc-tagged and untagged rescue constructs driving the expression of *ttd14*-A or *ttd14-*B in R1-6 photoreceptor cells under the control of the *Rh1* promoter. Upon expression of the constructs in *ttd14* ^*P75L*^ homozygous mutant eye clones, both *ttd14* isoforms rescued the TRPL trafficking defects (S1A Fig). In addition, water immersion microscopy analysis of homozygous mutant eye clones harboring the lethal P-element insertion *ttd14* ^*KG03769*^ in the *ttd14* gene revealed the same TRPL trafficking defect as was observed in the *ttd14* ^*P75L*^ mutant (S1B Fig). These results demonstrate that the lethal *ttd14* ^*P75L*^ mutation causes the TRPL trafficking defect in homozygous mutant eye clones.

### The *ttd14* gene encodes a cytosolic GTP and phospholipid-binding protein conserved among invertebrate species

The protein encoded by the *ttd14* gene will hereafter be referred to as TTD14. No functional data about TTD14 are available so far. Protein domain prediction revealed a P-loop-containing nucleoside triphosphate hydrolase domain (P-loop) and a CYTH-like domain (Fig 2A). The predicted P-loop of TTD14 contains a *bona fide* GxxxxGKT Walker A motif (amino acids 73–80) and a possible hhhhDxG Walker B motif at position 152–158 (canonical Walker B motif: hhhhDxxG, where h is a hydrophobic and x is any amino acid; [40–42]). The mutated amino acid P75 is located in the Walker A motif and is highly conserved in the TTD14 homologs of other invertebrate species. CYTH domains were identified in bacterial adenylyl cyclases and mammalian thiamine triphosphatases [43]. Although highly conserved orthologs of TTD14 are present in other invertebrates such as bees or *C*. *elegans* (Fig 2B), BLAST sequence similarity searches did not reveal full-length homologs in vertebrates with significant sequence similarity to TTD14.

**Fig 3:**
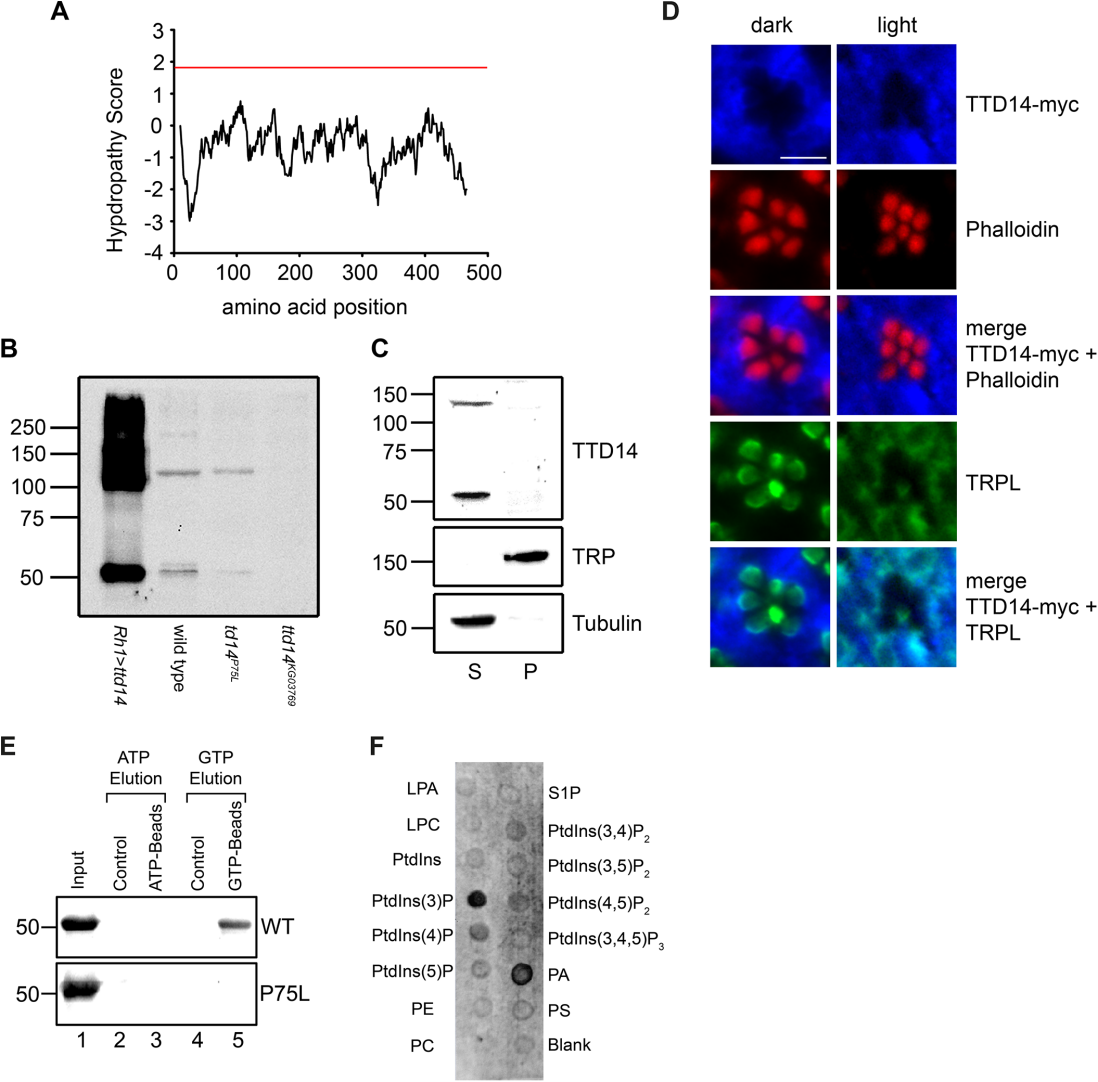
Characterization of the TTD14 protein. (A) Kyle-Doolittle Hydropathy Plot (http://web.expasy.org/protscale, window size 19) of the TTD14-A protein. The red line denotes a lower limit for transmembrane helices. (B) Immunoblot of protein extracts obtained from dissected eye cups (equivalent of 12 eyes per lane) of flies overexpressing TTD14-A under the control of the Rh1 promoter (*Rh1*>*ttd14-A*), wild type flies and from mosaic eyes composed of *ttd14* ^*P75L*^ or *ttd14* ^*KG03769*^ homozygous mutant cell clones (80–90%) and heterozygous clones (10–20%). In wild type the anti-TTD14 antibody detected two bands at 55 kDa and 130 kDa that were reduced or absent in *ttd14* ^*P75L*^ or *ttd14* ^*KG03769*^ mutant eyes. In extracts of *Rh1>ttd14-A* eyes these bands were greatly enhanced and additional labeling was observed at higher molecular weight regions. The size of molecular weight markers in kilo Dalton is indicated at the left. (C) Immunoblot of protein extracts from *Drosophila* heads (equivalent of 6 or 12.5 heads for supernatant and pellet, respectively) showing the distribution of the TTD14 protein between the soluble supernatant (S) and the membrane pellet (P). TRP and Tubulin were used as an example for membrane and soluble proteins, respectively. The size of molecular weight markers in kilo Dalton is indicated at the left. (D) Immunocytochemical staining of myc-tagged TTD14 transgenically expressed under the control of the Rh1-promoter (TTD14-myc) in 3–5 day old wild type flies exposed to darkness (dark) or orange light (light) for 16 hours. Cross sections of ommatidia were probed with a monoclonal anti-myc antibody (blue, first row) and Alexa Fluor 546-conjugated-phalloidin (red, second row). Alexa Fluor 546-conjugated phalloidin labels the actin cytoskeleton of rhabdomeres. A merged image is shown in the third row. Co-localization of TTD14-myc (blue, first row) and TRPL (green fourth row) is shown in the lowest row. Scale bar: 5 μm. (E) *In vitro* nucleotide binding assay. 20 μg of purified His-tagged TTD14-A protein (WT, upper panel) and purified His-tagged TTD14-A protein containing the P75L mutation (P75L, lower panel) were incubated with 25 μl control agarose (lanes 2, 4), ATP agarose (lane 3) or GTP agarose (lane 5). 2 μg of the purified TTD14 proteins were loaded in lane 1. After washing away unbound proteins, TTD14 was eluted from the beads using either ATP (lanes 2, 3) or GTP (lanes 4, 5) and subjected to SDS-PAGE, followed by immunoblot detection using the anti-TTD14 antibody. (F) Phospholipid binding assay. Purified His-tagged TTD14-A (0.4 μg/ml) was incubated with nitrocellulose strips (PIP-Strips) spotted with 100 pmol of each of the indicated phospholipids (LPA: Lysophosphatatic acid, LPC: Lysophosphocholine, PE: Phosphatidylethanolamine, PC: Phosphatidylcholine, S1P: Sphingosine-1-phosphate, PA: Phosphatidic acid, PS: Phosphatidylserine). Binding of TTD14-A was detected with the anti-TTD14 antibody.

Hydrophobicity analysis of TTD14 suggested that the protein is a soluble or a peripheral membrane protein since no sufficiently hydrophobic regions for putative transmembrane domains were predicted (Fig 3A). In order to determine the subcellular localization of TTD14 experimentally, we performed biochemical fractionation experiments and immunocytochemistry. We generated a polyclonal antibody against a recombinantly-expressed full length TTD14-A protein. To test the specificity of the generated antibody, an immunoblot experiment was carried out using protein extracts of dissected *Drosophila* eyes of wild type flies, of flies overexpressing TTD14-A in photoreceptor cells under the control of the Rh1 promoter, and of flies having homozygous mutant *ttd14* eye clones (comprising 80–90% of the eye) (Fig 3B). The anti-TTD14 antibody detected two protein bands with an apparent molecular weight of 55 kDa and 130 kDa, respectively, in wild type eyes. These bands were reduced or undetectable in *ttd14* ^*P75L*^ and *ttd14* ^*KG03769*^ mosaic eyes, respectively, and greatly enhanced in eyes overexpressing TTD14-A. Although it cannot be excluded that residual TTD14 protein detected in eyes with *ttd14* ^*P75L*^ mutant eye clones results from the eye parts that are heterozygous for *ttd14* ^*P75L*^, the loss of TTD14 in these eyes seems to be less severe than in *ttd14* ^*KG03769*^ mosaic eyes. Therefore, the P75L point mutation may less severely affect the stability of the TTD14 protein but rather impair protein function. Since the calculated molecular weight is 54,3 kDa for TTD14-A and 53,5 kDa for TTD14-B, we assume that the 55 kDa and 130 kDa bands detected by the antibody represent monomers and dimers of TTD14-A and -B isoforms.

For fractionation experiments, *Drosophila* heads were homogenized in Tris/NaCl-buffer and soluble and membrane proteins were separated by ultracentrifugation (Fig 3C). Using immunoblot analysis, the TTD14 protein was detected exclusively in the soluble fraction. Although the antibody specifically detected TTD14 on immunoblots, it did not yield specific signals when used in immunocytochemistry. Therefore, we used flies expressing a myc-tagged TTD14 (TTD14-myc) in photoreceptor cells under the control of the *Rh1* promoter and anti-myc antibodies for immunocytochemistry on cross sections through eyes of 24 hours dark- or 16 hours orange light-adapted flies. These experiments revealed a rather uniform signal in the cell body but no signal in the rhabdomeres (Fig 3D). No obvious differences in localization of TTD14-myc between photoreceptor cells of light- or dark-adapted flies were observed. Co-localization of TRPL and TTD14-myc on cross sections through eyes showed that there is indeed a considerable overlap in TRPL and TTD14-myc staining in eyes of light-adapted flies but not in eyes of dark-adapted flies where TRPL is localized in the rhabdomeres (Fig 3D). It has to be noted, however, that in eyes of light-adapted flies, TTD14-myc staining does not completely overlap with TRPL staining, suggesting that the TTD14 protein is not specifically enriched in membrane compartments occupied by TRPL.

In order to test the predicted nucleotide binding activity of the TTD14 protein, we performed an *in vitro* nucleotide binding assay. Purified recombinant full-length TTD14-A protein was incubated with agarose beads coupled to ATP, GTP, or no nucleotide (control). After removal of unbound protein, TTD14 was eluted from the beads with either 25 mM ATP or 25 mM GTP. TTD14 protein was recovered from GTP beads but not from ATP or control beads indicating that TTD14 is a GTP-binding protein (Fig 3E). In order to test an effect of the P75L mutation present in the *ttd14* ^*P75L*^ allele on GTP-binding, we generated a plasmid for recombinant expression of TTD14 protein harboring the P75L amino acid substitution. This amino acid substitution did not affect protein stability as the mutated protein could be purified in an amount comparable to the wild type TTD14 protein (Fig 3E). However, the P75L mutation abolished GTP binding of TTD14 in the *in vitro* nucleotide binding assay (Fig 3E) indicating that the predicted Walker A motif (amino acids 73–80) is likely to be involved in GTP binding. In addition, the mutant phenotype of the *ttd14* ^*P75L*^ allele might be attributed to the loss of GTP binding activity, suggesting that GTP binding is essential for the biological function of the TTD14 protein.

As differentially-distributed phosphoinositides play a crucial role in membrane trafficking by recruiting trafficking proteins to the membrane [44], we tested a possible interaction of TTD14 with membrane lipids. In a lipid binding assay we employed nitrocellulose strips spotted with 100 pmol of various phospholipids (PIP-Strips). The PIP-Strips were incubated with purified recombinant full length TTD14-A protein and bound protein was detected with the anti-TTD14 antibody. As a result, among the 15 lipids tested, TTD14 bound to 3-phosphoinositide (PtdIns(3)P) and to phosphatidic acid (PA) (Fig 3F). PtdIns(3)P is predominantly localized at the cytosolic side of membranes of early endosomes [44], suggesting that TTD14 interacts with early endosome membranes.

Collectively, our results reveal that TTD14 is a soluble, most likely cytosolic protein expressed in the *Drosophila* eye that binds GTP and interacts with PtdIns(3)P and PA.

### TTD14 is required for trafficking of the TRPL ion channel between rhabdomere and cell body

**Fig 4:**
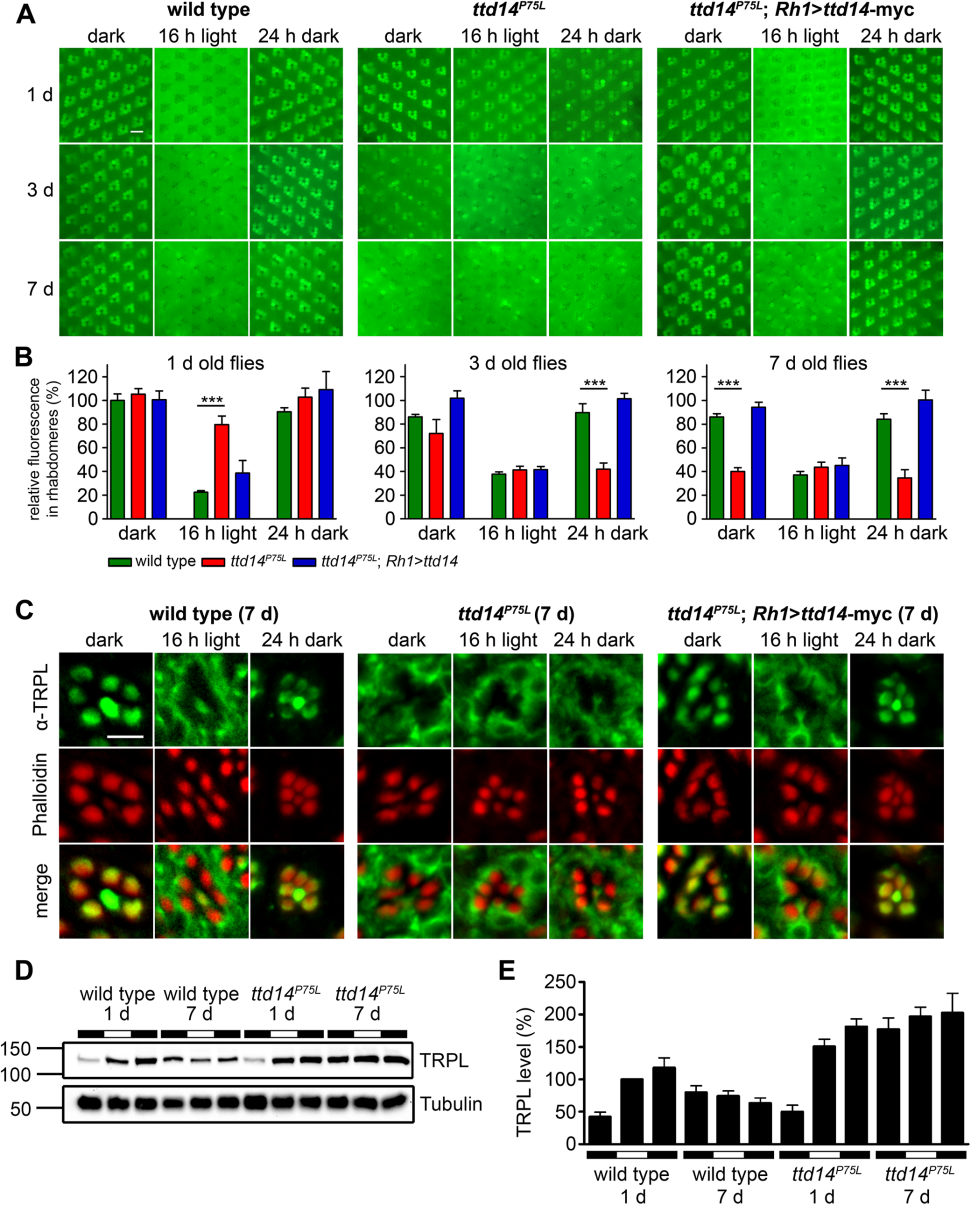
Characterization of the TRPL trafficking defect in the *ttd14* ^*P75L*^ mutant. (A) Water immersion microscopy images of TRPL-eGFP fluorescence in white colored (*white* ^*–*^) eyes of wild type flies (wild type), *ttd14* ^*P75L*^ mutant eye clones (*ttd14* ^*P75L*^), and flies expressing a *ttd14-myc* construct under the control of the Rh1 promoter in *ttd14* ^*P75L*^ mutant eye clones (*ttd14* ^*P75L*^ *; Rh1*>*ttd14-A-myc*). After eclosion, flies were kept in the dark for 1 day (upper row), 3 days (middle row) or 7 days (lower row) and subsequently exposed to orange light for 16 hours. After orange light illumination, flies were subjected to a second dark-adaptation for 24 hours. Localization of TRPL-eGFP in rhabdomeres or in the cell body appears, respectively, as distinct circular signal or as a diffuse signal with dark rhabdomeres. Scale bar: 10 μm. (B) Quantification of the relative fluorescence of TRPL-eGFP in rhabdomeres of the outer photoreceptor cells using water immersion images as shown in (*A*) (mean ± SD; n = 5). The relative rhabdomeral TRPL-eGFP fluorescence was determined as described in Material and Methods and the value obtained for wild type flies kept in darkness for 1 day was set to 100%. Statistically significant differences between wild type and *ttd14* ^*P75L*^ as analyzed by an unpaired Student´s *t* test are indicated (***, p<0.001). (C) Localization of native TRPL on cross sections through ommatidia from wild type flies, *ttd14* ^*P75L*^ mutant eye clones and *ttd14* ^*P75L*^ mutant eye clones expressing a *Rh1*>*ttd14-A-myc* construct (*ttd14* ^*P75L*^ *; Rh1*>*ttd14-A-myc*). Flies were aged for 7 days in darkness and subsequently subjected to the same light-regime as in (A). Cross sections were probed with an anti-TRPL antibody (green, upper row) and Alexa Fluor 546-coupled phalloidin (red, middle row). Merged panels are shown in the lower row. Scale bar: 5 μm. (D) Immunoblot analysis of TRPL extracted from wild type heads or from heads with *ttd14* ^*P75L*^ mutant eye clones (equivalent of 3 heads per lane). Freshly eclosed flies (1 day) or flies kept in the dark for 7 days were analyzed immediately (first black bars) or subjected to orange light illumination for 16 hours (white bars) followed by 24 hours of darkness (second black bars). The blots were probed with α-TRPL and α-Tubulin antibodies as indicated. The size of molecular weight markers in kilo Dalton is indicated at the left. (E) Quantification of the TRPL levels normalized to Tubulin. The TRPL level of 1 day old flies illuminated for 16 hours (second column) was set to 100%. Error bars show SEM (n = 5).

For a detailed analysis of the TRPL trafficking defect in *ttd14* ^*P75L*^, flies expressing TRPL-eGFP in R1-6 photoreceptor cells were subjected to water immersion microscopy of intact eyes and the TRPL-eGFP fluorescence in rhabdomeres was determined as in [35] (Fig 4A). As the generated *Rh1*>*ttd14-A* and *Rh1*>*ttd14-B* rescue constructs are associated with a *white* ^*+*^-marker and result in an orange eye color that interferes with the quantification of water immersion images, a *yellow* ^+^-marked *Rh1*>*ttd14-A-myc* construct bearing a C-terminal myc-tag was expressed in white-eyed flies and used for this quantitative analysis. Qualitatively, rescue of the *ttd14* ^*P75L*^ phenotype by untagged *ttd14* constructs is shown in S1A Fig. In addition, the phenotype in homozygous *ttd14* ^*KG03769*^ mutant eye clones, that have red eye color, is depicted in S1B Fig and compared to red-eyed wild type flies.

In wild type flies (Fig 4B green bars), long-term dark incubation for up to 7 days only slightly reduced the rhabdomeral TRPL-eGFP fluorescence. In contrast, after 16 hours illumination with orange light, rhabdomeral fluorescence was reduced by 78% in 1 day old flies, indicating that most of TRPL-eGFP had translocated to the cell body. Light-triggered TRPL translocation was less efficient in flies that were previously kept in darkness for 3 days or 7 days but still resulted in a reduction of the rhabdomeral TRPL-eGFP fluorescence by more than 55%. Subsequent dark adaptation for 24 hours fully restored the original rhabdomeral TRPL-eGFP fluorescence irrespective of the duration of the initial dark incubation.

In *ttd14* ^*ttd14*^ mutant eye clones (Fig 4B, red bars) of 1 day old flies, TRPL-eGFP was properly located in the rhabdomere in the dark, but hardly translocated to the cell body upon illumination with orange light for 16 hours. With increasing time of dark incubation, TRPL-eGFP fluorescence progressively disappeared from the rhabdomere resulting in a rhabdomeral TRPL-eGFP fluorescence of only 40% in 7 day old flies. Orange light illumination in these flies did not further reduce the rhabdomeral TRPL-eGFP content. Of primary significance here, subsequent dark adaptation for 24 hours did not affect TRPL-eGFP distribution in 3 day or 7 day old flies, indicating that recycling of TRPL-eGFP to the rhabdomere was severely impaired. This latter phenotype is less prominent in young flies, as the initial rhabdomeral TRPL content before dark adaptation is already elevated. In conclusion, mutation of the *ttd14* gene did not affect the initial localization of TRPL-eGFP in the rhabdomere but resulted in reduced transport of TRPL-eGFP from the rhabdomere to the cell body in young flies. Upon prolonged incubation of flies in the dark, net transport of TRPL-eGFP from the cell body to the rhabdomere was reduced as most of TRPL-eGFP was located in the cell body and failed to recycle to the rhabdomere irrespective of the light condition. All aspects of the mutant phenotype described above were rescued by expression of *ttd14* wild type constructs (Fig 4B, blue bars and S1A Fig) in R1-6 photoreceptor cells under control of the *Rh1* promoter showing that TTD14 function is required in photoreceptor cells.

In order to reveal the subcellular localization of TRPL with higher resolution, we performed immunocytochemistry of 7 day old wild type, *ttd14* ^*P75L*^, and rescue flies (Fig 4C). Like in the water immersion experiments, the flies were first kept in constant darkness, then illuminated with orange light for 16 hours and then again kept in darkness for 24 hours. As observed previously [34, 37], wild type flies revealed TRPL labeling in the rhabdomeres when kept in darkness and a relatively uniform labeling of the cell bodies (except for nuclei) when kept in light. The same labeling pattern was observed in *ttd14* mutant eye clones of flies expressing the rescue construct Rh1>*ttd14*-myc. Without the rescue construct, *ttd14* mutant eye clones revealed a TRPL labeling pattern in the cell body in all light and dark conditions tested that was indistinguishable from that of wild type flies kept in the light. We previously reported a localization defect of a mutated TRPL, in which eight C-terminal phosphorylation sites were abolished [45]. However, the mislocalization of phosphorylation-deficient TRPL resulted in labeling of distinct spots in the cell body quite different from the uniform TRPL labeling pattern observed in the *ttd14* mutant (S2 Fig and see Ref. 45). In addition to the localization defect, removal of TRPL phosphorylation sites affected TRPL stability in the dark and resulted in progressive TRPL degradation [45]. To assay TRPL stability in the *ttd14* ^*P75L*^ mutant, we carried out immunoblot analyses with protein extracts obtained from 1 day and 7 day old wild type flies and from the *ttd14* ^*P75L*^ mutant subjected to different light conditions (Fig 4D and 4E). A lower amount of TRPL in 1 day old flies, which were assayed immediately (that is before further incubation in light or darkness, Fig 4D, lanes 1 and 7), was observed in both wild type and mutant, suggesting that the TRPL content in freshly eclosed flies is lower than in older flies. No indication of TRPL degradation in the *ttd14* ^*P75L*^ mutant flies was observed. Indeed, except for flies freshly eclosed in the dark (Fig 4E, first and 7^th^ column), *ttd14* ^*P75L*^ mutant flies exhibited significantly (p < 0.05) higher TRPL protein levels as compared to the corresponding condition in wild type flies. Thus, TRPL in the *ttd14* ^*P75L*^ mutant is not targeted to the lysosomal degradation pathway. It rather seems that the *ttd14* mutation affects the recycling of TRPL which accumulates in the internal storage compartment.

### TTD14 is not required for Rh1 or TRP trafficking

**Fig 5:**
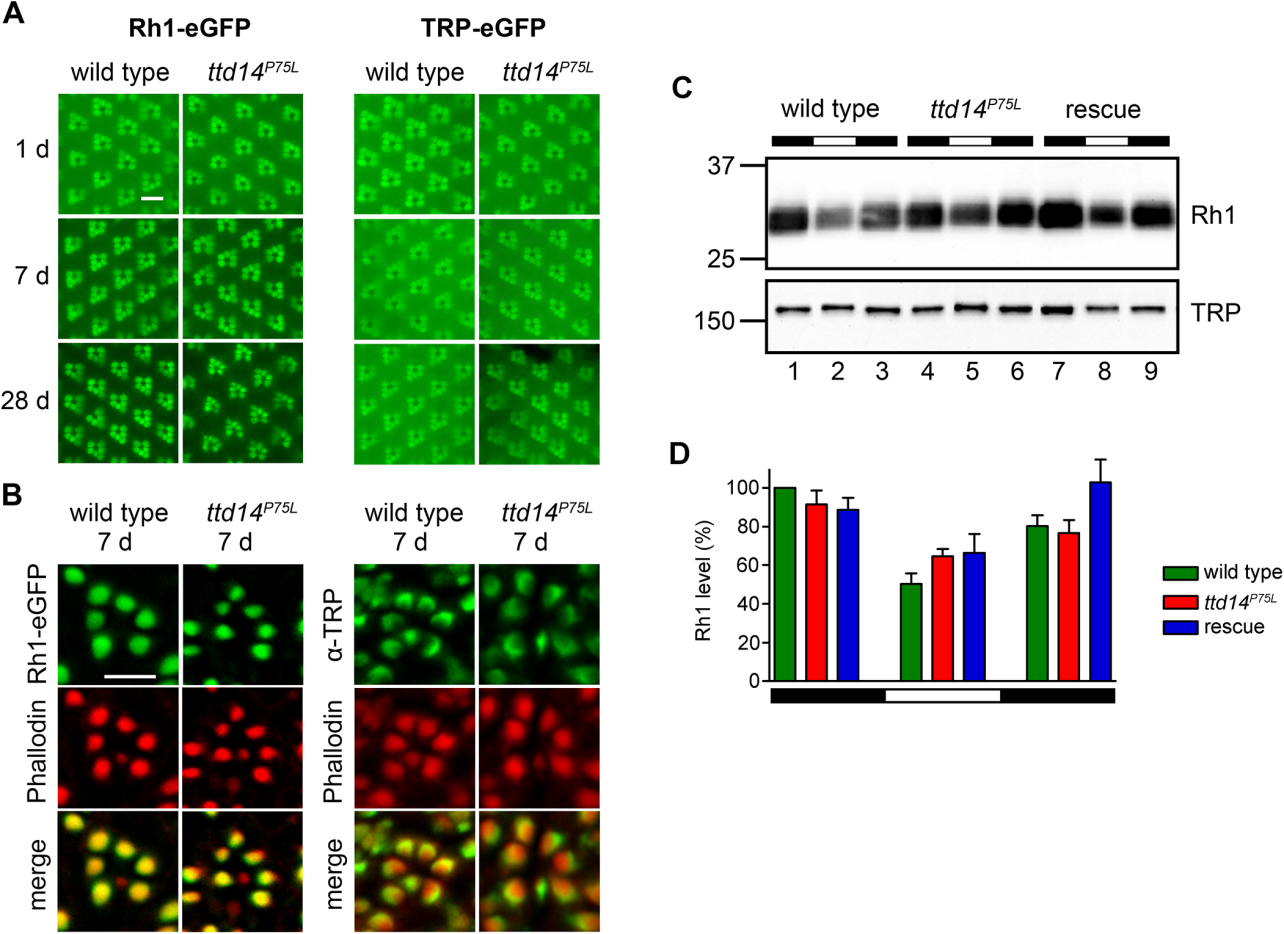
Rhabdomeral localization of Rh1 and TRP is not affected in the *ttd14* ^*P75L*^ mutant. (A) Water immersion microscopy images of the Rh1-eGFP and TRP-eGFP fluorescence in the eyes of wild type flies (wild type) and *ttd14* ^*P75L*^ mutant eye clones (*ttd14* ^*P75L*^). Flies were kept in constant darkness for 1 day (upper row), 7 days (middle row) or 28 days (lower row). Scale bar: 10 μm. (B) Analysis of Rh1-eGFP and TRP localization on cross sections through ommatidia of wild type flies (wild type) and *ttd14* ^*P75L*^ mutant eye clones (*ttd14* ^*P75L*^), kept in darkness for 7 d. Rh1-eGFP localization was detected by its eGFP fluorescence (green, upper row), TRP localization was detected by an anti-TRP-antibody (green, upper row). The actin cytoskeleton of rhabdomeres was labeled with Alexa Fluor 546-coupled phalloidin (red, middle row). Overlay of red and green fluorescence appears yellow in the merged panels. Scale bar: 5 μm. (C) Immunoblot assessing Rh1 and TRP from heads of wild type flies (wild type, lanes 1–3), *ttd14* ^*P75L*^ mutant flies (*ttd14* ^*P75L*^, lanes 4–6) and *ttd14* ^*P75L*^ *; Rh1*>*ttd14-myc* flies (rescue, lane 7–9). Protein from 0.5 heads was loaded per lane. Flies were kept in darkness (lanes 1,4,7), exposed to white light (1800 lux) for 20 hours (lanes 2,5,8), or exposed to white light for 20 hours followed by a 6 hour recovery in the dark (lanes 3,6,9). The size of molecular weight markers in kilo Dalton is indicated at the left. (D) Quantification of the Rh1 levels normalized to TRP. Error bars show SEM (n = 4). Rh1 levels decrease after illumination due to lysosomal degradation of internalized Rh1. No significant differences were found between wild type, *ttd14* ^*P75L*^ mutant and rescue flies at any light condition.

While TRPL becomes internalized from the rhabdomere in the light and is recycled back to the rhabdomere in the dark, two other important *Drosophila* photoreceptor membrane proteins, Rh1 and TRP, do not seem to undergo such a regulated change in their subcellular localization. Rh1 is constantly renewed in illuminated photoreceptor cells as the internalized Rh1 becomes partially degraded in the lysosome and rhabdomeral Rh1 is replenished by newly synthesized protein [20]. In addition, Rh1 is also partially recycled via the retromer complex [23]. However, both mechanisms do not result in a major change of the subcellular localization, as most of the Rh1 protein remains localized in the rhabdomere. Albeit less well studied, there is no evidence for a light-dependent translocation of the TRP protein. Indeed, a number of mutations in chaperones and transport proteins have been described that affect the anterograde transport of both Rh1 and TRP, but not TRPL suggesting a common transport pathway for these two photoreceptor proteins and a different pathway for TRPL (see introduction). Assuming that loss of functional TTD14 does not induce a general cytological defect but a specific defect in the internalization and recycling of membrane proteins such as TRPL, one might expect that the *ttd14* mutation does not affect trafficking of Rh1 and TRP. To test this assumption, we carried out water immersion microscopy with flies expressing eGFP-tagged Rh1 and TRP in R1-6 photoreceptor cells and also performed immunocytochemical experiments (Fig 5). While we had observed that TRPL-eGFP disappears from the rhabdomeres of *ttd14* mutants upon dark incubation for 7 days (see Fig 4), no age-related changes in the rhabdomeral content of Rh1-eGFP or TRP-eGFP were observed in water immersion microscopy for up to 28 days in darkness. The fluorescence pattern of Rh1-eGFP or TRP-eGFP expressed in *ttd14* ^*P75L*^ mutant eye clones was the same as in wild type (Fig 5A). Likewise, immunocytochemistry of cross sections through ommatidia of 7 day old flies kept in the dark, revealed no signs of mislocalization of Rh1-eGFP or native TRP that were both confined to the rhabdomeres in wild type flies as well as in *ttd14* ^*P75L*^ mutant eye clones (Fig 5B). In addition, we assayed the Rh1 and TRP protein content of 1 day and 7 day old *ttd14* ^*P75L*^ mutant flies, which were kept in darkness and then subjected to orange light illumination for 16 hours, by immunoblot analysis and observed the same protein content as in wild type flies (S3 Fig, Fig 4). Taken together, these results strongly suggest that the transport of Rh1 or TRP to the rhabdomere is not affected in the *ttd14* ^*P75L*^ mutant. Internalization of Rh1 can be assessed by immunoblot analysis when flies are illuminated with bright white light for at least 16 hours. In wild type flies, this illumination results in a reduced Rh1 level due to lysosomal degradation and the original Rh1 level is recovered after a subsequent incubation in darkness for 6 hours [23]. In mutants with defective Rh1 internalization, reduced lysosomal degradation would be expected. In contrast, in mutants affecting components required for Rh1 recycling like the retromer complex, enhanced Rh1 degradation results in a stronger reduction of the Rh1 level as compared to the wild type [23]. In illuminated *ttd14* ^*P75L*^ mutant flies, the Rh1 level was reduced to a similar degree as in wild type flies (Fig 5C and 5D) arguing against a major role of the TTD14 protein in Rh1 trafficking.

**Fig 6:**
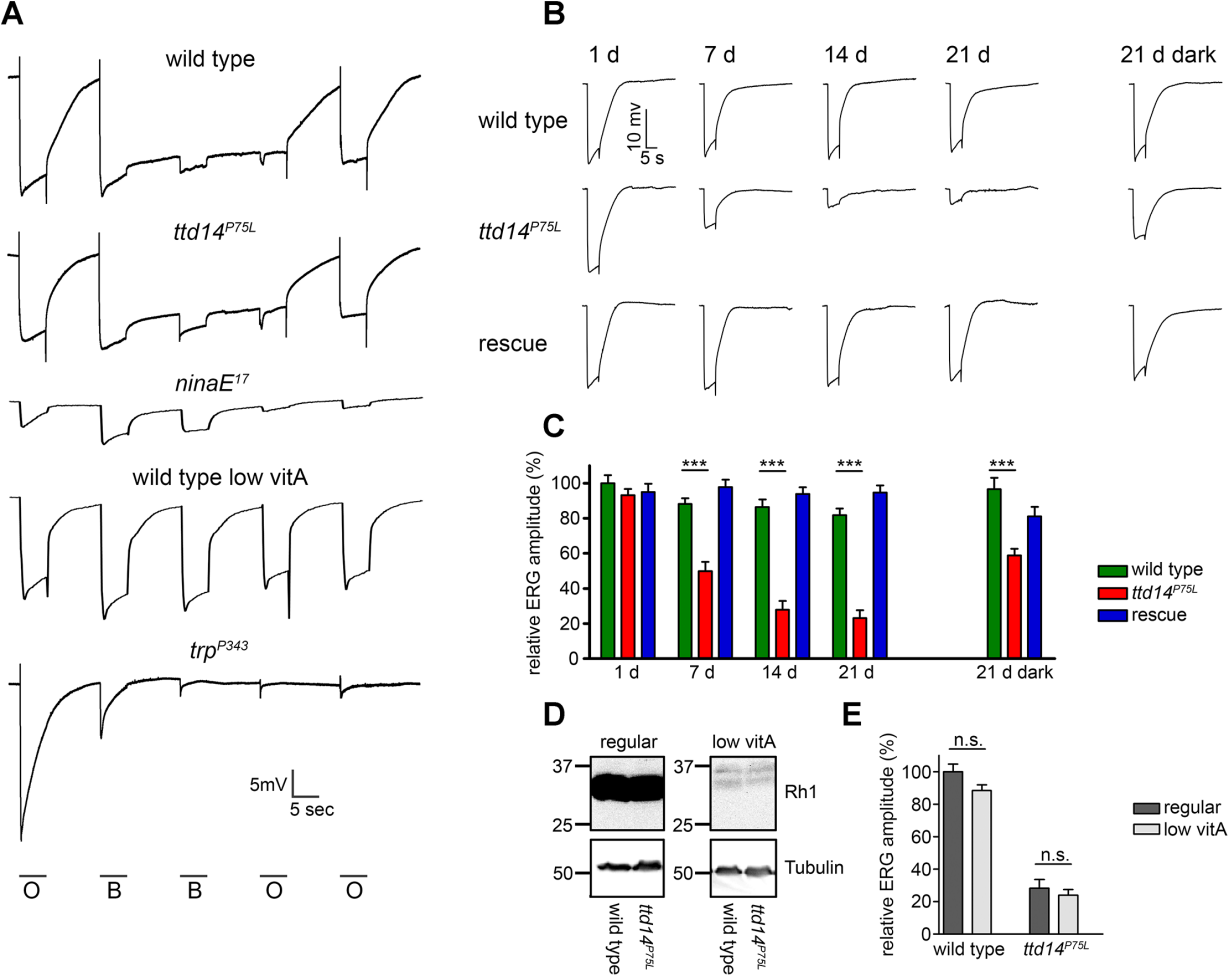
Aged *ttd14* ^*P75L*^ mutant flies kept in a 12 hours light/ 12 hours dark cycle develop physiological defects. (A) Electroretinogram recordings from 7 day old wild type and *ttd* ^*P75L*^ mutant flies, from 1 day old *ninaE* ^*17*^ mutant, 14 day old wild type flies raised on vitamin A-deprived food, and 1 day old *trp* ^*P343*^ mutant. All flies were raised and kept in the dark. Flies were stimulated with orange light (O) or blue light (*B*) for 5 seconds with dark intervals of 10 seconds between the stimuli. (B) ERG recordings from wild type flies, *ttd14* ^*P75L*^ mutant flies and *ttd14* ^*P75L*^ *; Rh1*>*ttd14-A-myc* flies (rescue) kept in a 12 hours light / 12 hours dark cycle for 1, 7, 14 and 21 days as well as after keeping the flies for 21 days in darkness (21 d dark, ERG traces at the right). Flies were stimulated with a 5 sec orange light pulse. (C) Quantification of the ERG amplitudes of flies kept in a 12 hours light / 12 hours dark cycle or in constant darkness as in *B*. Error bars represent SEM (n = 10). Statistically significant differences between wild type and *ttd14* ^*P75L*^ as analyzed by an unpaired Student´s *t* test are indicated (***, p<0.001). (D) Immunoblot analysis assessing Rh1 in wild type heads and heads with *ttd14* ^*P75L*^ mutant eye clones of flies kept in the dark for 14 days on either regular food (regular) or on a vitamin A-deprived diet (low vitA). The equivalent of 4 heads was loaded per lane. Tubulin was used as a loading control. Vitamin A deprivation resulted in a strong reduction of the Rh1 content. (E) Quantification of the ERG amplitude of 21 day old wild type and *ttd14* ^*P75L*^ mutant flies kept in a 12 hours light / 12 hours dark cycle. Flies were raised and kept either on regular food (regular) or on a vitamin A-deprived diet (low vitA). Error bars represent SEM (n = 10). Vitamin A deprivation did not affect the ERG amplitude as analyzed by an unpaired Student´s *t* test and did not rescue the reduced ERG amplitude of *ttd14* ^*P75L*^ mutant eyes.

Finally, in order to show that the *ttd14* mutant does not affect the function of Rh1 and TRP, we performed electroretinogram (ERG) recordings of wild type and *ttd14* ^*P75L*^ mutant flies kept in darkness for 7 d. At that time, TRPL was present in the rhabdomeres of wild type flies but not in the rhabdomeres of *ttd14* ^*P75L*^ mutant flies (Fig 4A–4C). A loss of rhabdomeral TRPL is not expected to have a major impact on the ERG, as the loss of TRPL in the *trpl* ^*302*^ null mutant, has an effect on light adaptation, but no impact on the shape of ERG recordings [46]. In contrast, impaired Rh1 or TRP function causes characteristic changes in ERG recordings (Fig 6A). A severe reduction in the amount of Rh1, which can be achieved by feeding flies with a vitamin A-deprived diet (see below), resulted in a loss of the prolonged depolarization afterpotential (PDA) after bright blue light illumination while a complete loss of Rh1, like in the *ninaE* ^*17*^ mutant, was readily indicated by a dramatic reduction of the ERG amplitude (Fig 6A). Loss of TRP function, like in the *trp* ^*P343*^ mutant, can be detected by the characteristic transient response to a light stimulus (Fig 6A). Using a stimulus protocol containing both orange and blue light illumination, we did not observe any obvious differences in ERG recordings from *ttd14* ^*P75L*^ mutant eye clones as compared to wild type (Fig 6A). We conclude that besides TRPL, neither Rh1 nor TRP nor any other major component of the phototransduction cascade is severely affected in *ttd14* ^*P75L*^ mutant flies.

### TTD14 is required for preventing photoreceptor degeneration

In order to asses long-term effects of the *ttd14* ^*P75L*^ mutation on photoreceptor function, we carried out ERG recordings of wild type flies, *ttd14* ^*P75L*^ mutant flies, and flies expressing the rescue construct *Rh1>ttd14-A-myc* in a *ttd14* ^*P75L*^ mutant background (Rescue) for up to 21 d. In a 12 hours light / 12 hours dark cycle, *ttd14* ^*P75L*^ mutant flies exhibited a decline in ERG amplitude after 7 days and an almost abolished photoresponse after 14 days (Fig 6B and 6C). After 21 days in constant darkness, *ttd14* ^*P75L*^ mutant flies also displayed a reduction in the ERG amplitude, albeit to a much lower extent than in a 12 hours light / 12 hours dark cycle (Fig 6B and 6C). In mutants of the retromer complex, photoreceptor degeneration that results in a loss of photoreceptor function is based on the mislocalization of Rh1 and can be attenuated by a reduction of the Rh1 level [23]. The Rh1 level can effectively be reduced by raising the flies on a vitamin A-deprived diet as the opsin protein becomes degraded in the absence of its chromophore (Fig 6D). In contrast to mutants of the retromer complex, diet-induced reduction of the Rh1 level had no significant effect on the ERG amplitude in response to an orange light stimulus in wild type or *ttd14* ^*P75L*^ mutant flies and did not rescue the declined ERG observed in *ttd14* ^*P75L*^ mutant flies kept in a 12 hours light / 12 hours dark cycle for 21 days (Fig 6E). In line with our results showing that the *ttd14* ^*P75L*^ mutation does not affect Rh1 localization (see Fig 5), this finding suggests that aberrant Rh1 localization is not causal for the loss of photoreceptor performance in this mutant.

**Fig 7:**
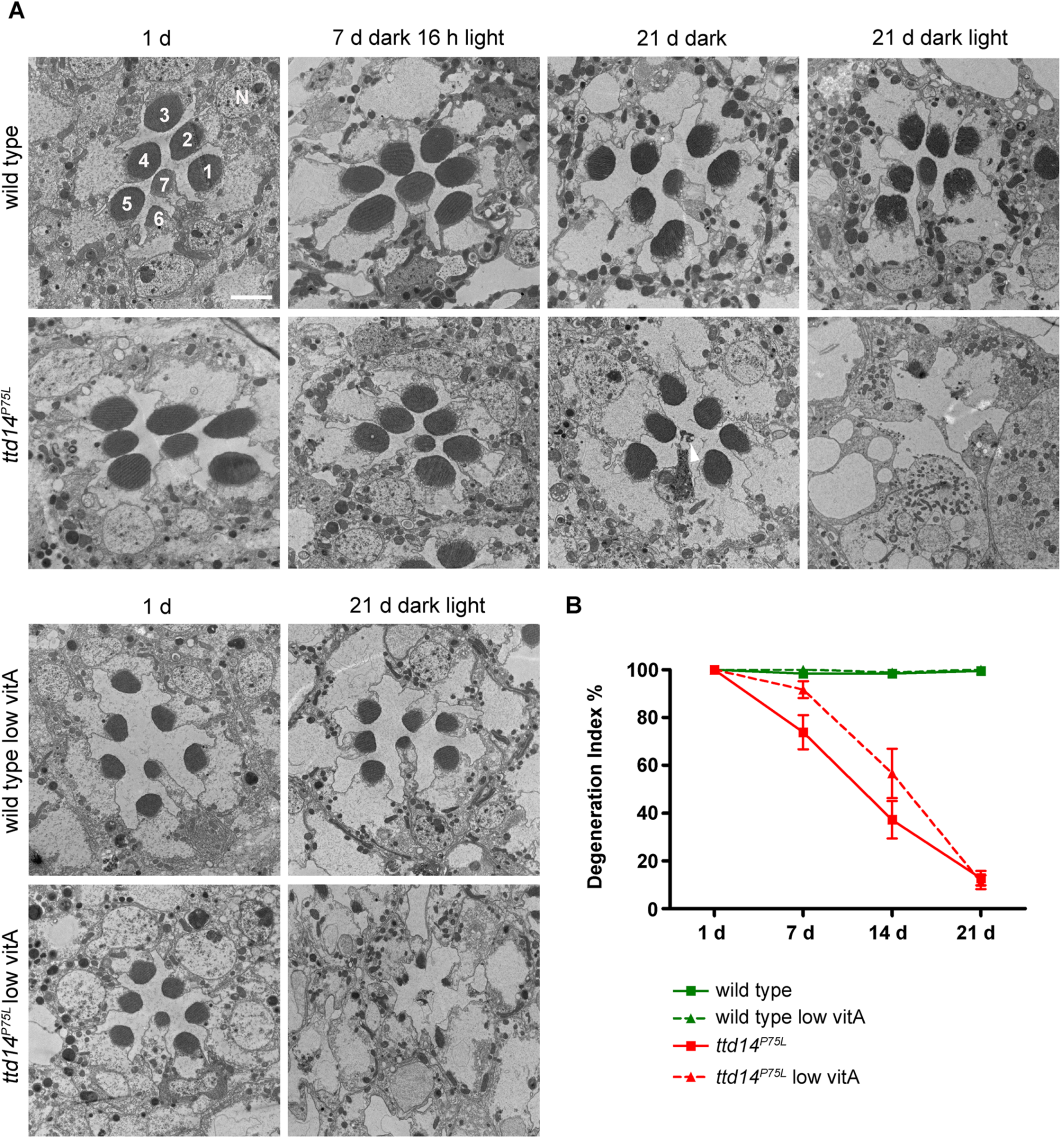
Photoreceptors of the *ttd14* ^*P75L*^ mutant undergo light-enhanced, but Rh1-independent degeneration. (A) Transmission electron microscopy of tangential sections through eyes of wild type flies and homozygous mutant eye clones of *ttd14* ^*P75L*^ mutant flies. Flies were assayed after eclosion (1 d), kept in the dark for 7 days followed by 16 hours orange light (7 d dark 16 h light), kept in the dark for 21 d, or subjected to a 12 hours light / 12 hours dark cycle for 21 d (21 d dark light). Flies were either raised on normal food (upper panels) or on vitamin A-deprived diet (low vitA; lower panels). 1–7 denotes rhabdomeres of photoreceptor cells R1 to R7. N, nucleus. Scale bar: 2.5 μm. Wild type flies display a normal morphology of the rhabdomeres at all conditions analyzed, except that vitamin A-deprived flies have smaller rhabdomeres due to the reduced amount of rhodopsin. Degeneration of inner and outer photoreceptor cells is obvious in *ttd14* ^*P75L*^ mutants exposed to a light/dark cycle for 21 days. Interestingly, R7 cells show signs of cell death while R1-6 cells are not affected (arrowhead) in *ttd14* ^*P75L*^ mutants kept in the dark for 21 days. (B) Time course of photoreceptor degeneration in the *ttd14* ^*P75L*^ mutant raised on normal food or on vitamin A-deprived diet (low vitA). Fluorescent water immersion images of wild type flies and *ttd14* ^*P75L*^ mutant eye clones, which express TRP-eGFP as a fluorescent marker for rhabdomeres in photoreceptor cells R1-6, were scored for the presence of rhabdomeres (see Material and Methods). 100% represents fully intact rhabdomeres. 3 ommatidia from five flies each were analyzed. Error bars denote SEM. Flies were kept in a 12 hours light / 12 hours dark cycle for the indicated number of days. Examples of original images used for this analysis are shown in S3 Fig.

In order to assess if the decline of the ERG amplitude was associated with morphological alterations in the rhabdomeres, we assessed rhabdomeral structure in wild type and *ttd14* ^*P75L*^ mutant eye clones for up to 21 days in a 12 hours light / 12 hours dark cycle by transmission electron microscopy and by monitoring TRP-eGFP fluorescence (Fig 7). While no obvious changes could be detected in wild type eyes, electron microscopy revealed severe degeneration of photoreceptor cells in *ttd14* ^*P75L*^ mutant flies kept in a 12 hours light / 12 hours dark cycle for 21 days. Most rhabdomeres of photoreceptor cells R1-6 and also the rhabdomere of R7 were absent in these flies. Degeneration was much less pronounced when the *ttd14* ^*P75L*^ mutant was kept in the dark for 21 days. In these flies the R7 cell was affected frequently while almost all rhabdomeres of R1-6 cells remained intact. These findings show that degeneration of photoreceptor cells in the *ttd14* ^*P75L*^ mutant is enhanced by light and affects inner and outer photoreceptor cells. Of note, no signs of degeneration of inner or outer photoreceptor cells were detected in *ttd14* ^*P75L*^ mutant flies kept in the dark for 7 days followed by 16 h orange light, a condition in which TRPL failed to recycle back to the rhabdomere. This finding suggests that the TRPL trafficking defect in the *ttd14* ^*P75L*^ mutant is not a result of photoreceptor cell degeneration. As observed before Vitamin A deprivation resulted in diminished rhabdomere size [23]. However, degeneration was also observed in vitamin A-deprived *ttd14* ^*P75L*^ mutant flies showing that a reduction of the rhodopsin content cannot rescue the degeneration phenotype. Using water immersion microscopy with *ttd14* ^*P75L*^ mutant eye clones that express TRP-eGFP as a rhabdomeral marker we analyzed the time course for the loss of rhabdomeres both under regular and vitamin A-deprived conditions (Fig 7B and S4 Fig). In flies kept in a light-dark cycle first signs of degeneration were detected after seven days while almost all rhabdomeres were lost after 21 days (Fig 7B). Vitamin A-deprivation had a small but not statistically significant effect on the degeneration time course and slightly slowed down the speed of degeneration in the *ttd14* ^*P75L*^ mutant. Taken together, loss of functional TTD14 in the *ttd14* ^*P75L*^ mutant resulted in late onset light-dependent, but Rh1-independent retinal degeneration.

## Discussion

Recycling of plasma membrane proteins plays a pivotal role in the function and maintenance of neurons. Defects in protein recycling caused, for example, by mutations in components of the retromer complex that regulates recycling and retrograde transport of proteins in early endosomes have been implicated in detrimental neurodegenerative diseases such as Alzheimer’s disease, Parkinson’s disease and Down´s syndrome [32, 47–49]. The TRPL ion channel of *Drosophila* photoreceptor cells is a useful model for studying recycling of neuronal plasma membrane proteins as its internalization from and redistribution to the plasma membrane can be triggered simply by exposing the flies to light or darkness, respectively [34–36, 45]. Due to its genetic versatility, *Drosophila* offers the possibility to identify novel components of protein recycling pathways in neurons by unbiased genetic screens. We had previously performed a genetic screen for mutants defective in TRPL ion channel transport [39] and in this study, we identified the orphan gene *ttd14* as a major regulator of TRPL trafficking in *Drosophila* photoreceptor cells.

### The *ttd14* mutation blocks TRPL recycling

**Fig 8:**
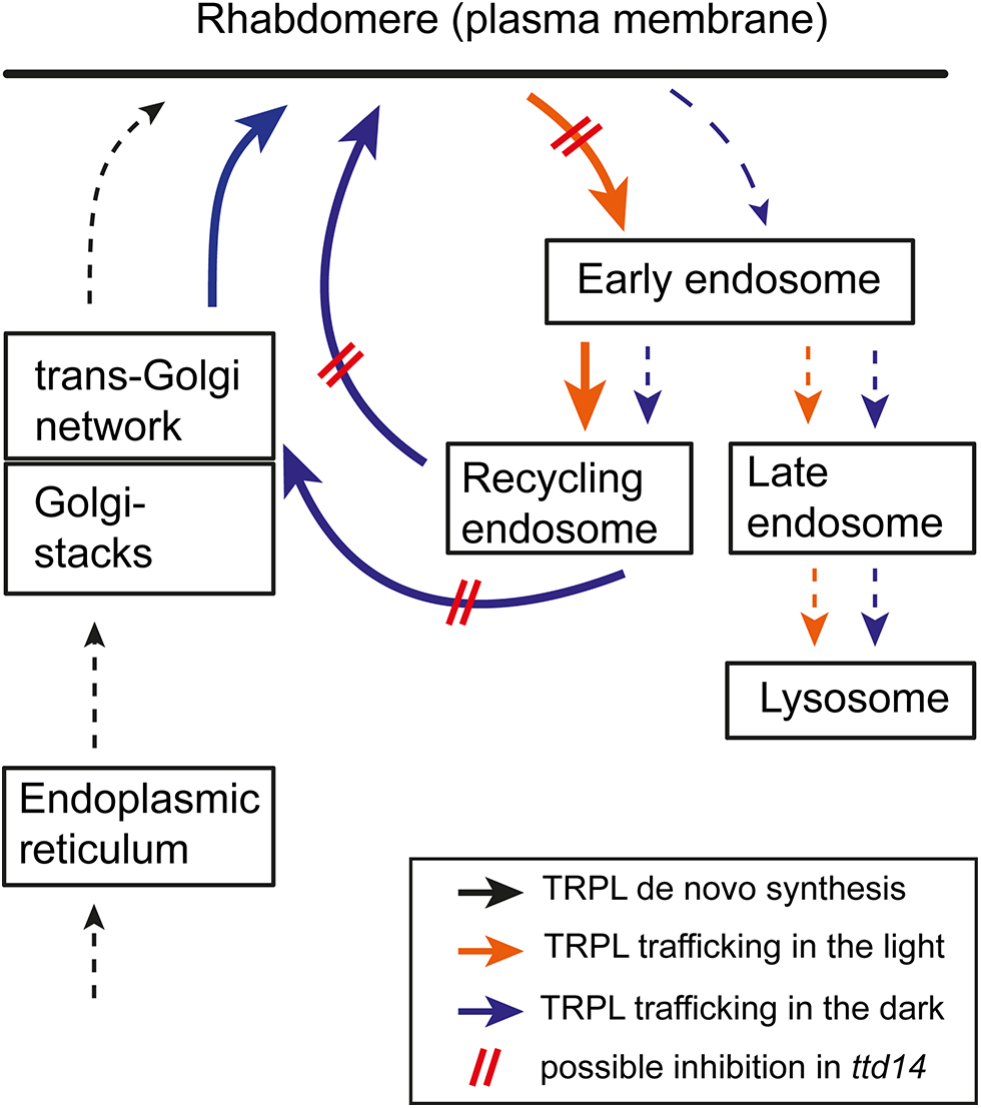
Proposed scheme of TRPL trafficking. Trafficking routes of TRPL in illuminated photoreceptors are illustrated with orange arrows while trafficking routes of TRPL in dark-kept photoreceptors are shown by blue arrows. De novo synthesis of TRPL is indicated by black arrows. Thick arrows and broken arrows indicate major and minor trafficking routes, respectively. Disturbed trafficking of TRPL in *ttd14* mutant photoreceptors is illustrated by red bars.

The mutant *ttd14* ^*P75L*^ was initially identified by the internalization defect of TRPL-eGFP after illumination. However, TRPL internalization is not completely abolished in *ttd14* ^*P75L*^ mutant flies suggesting that the TTD14 protein is not crucial for TRPL internalization but rather acts as a modulator that facilitates TRPL internalization. Alternatively, TTD14 might play an indirect role in TRPL internalization and promote TRPL internalization by enabling recycling of a rhabdomeral protein that is required for TRPL internalization. The most prominent phenotype of the *ttd14* ^*P75L*^ mutant is the failure of TRPL trafficking from the storage compartment back to the rhabdomere during dark adaptation. This phenotype is not evident in young flies, in which TRPL is localized in the rhabdomeres, suggesting that delivery of newly synthesized TRPL to the rhabdomere via the secretory pathway is not affected by the *ttd14* ^*P75L*^ mutation. However, when TRPL is redistributed from the rhabdomere to the storage compartment in illuminated 3 day or 7 day old *ttd14* ^*P75L*^ mutant flies, subsequent dark adaptation for 24 hours does not result in redistribution of TRPL to the rhabdomere (see Fig 4). This finding indicates that internalized TRPL fails to become recycled to the plasma membrane in *ttd14* ^*P75L*^. Continuous dark adaptation of the *ttd14* ^*P75L*^ mutant for 7 days also results in localization of TRPL in the cell body. This finding indicates that there is a basal level of TRPL internalization in the dark. In wild type flies, TRPL that becomes internalized in the dark is readily recycled back to the rhabdomere resulting in little if any TRPL in the cell body in the dark. In *ttd14* ^*P75L*^, where TRPL recycling is blocked, prolonged dark adaptation depletes TRPL from the rhabdomere and results in TRPL accumulation in the cell body. We had previously observed that mutation of C-terminal phosphorylation sites of TRPL also results in depletion of mutated TRPL from the rhabdomere when the flies are kept in darkness for five days [45]. This finding again argues for a basal rate of TRPL internalization in the dark. The phosphorylation-deficient TRPL, however, does not accumulate in the cell body but becomes degraded. Therefore, this mutation might shift the balance between recycling and lysosomal degradation of TRPL towards degradation rather than hindering TRPL entry into the recycling pathway. The proposed cellular trafficking pathways of TRPL in light and darkness and the steps that are presumably disturbed in the *ttd14* mutant are illustrated in Fig 8.

### Biochemical properties of TTD14

Our biochemical analysis revealed that TTD14 is a soluble protein that binds GTP and the phospholipids PtdIns(3)P and phosphatidic acid (see Fig 3). Protein domain searches revealed a P-loop nucleotide binding domain comprising amino acids 65–167 of TTD14. This domain contains a bona fide Walker A and a possible Walker B motif and thus represents a likely site for binding of GTP or ATP. When compared with vertebrate proteins the domain exhibited 37% amino acid identity with the respective domain of mitochondrial GTPase Era (S1 Table). Binding assays with recombinant TTD14 revealed binding to GTP but not to ATP. The P75L mutation of *ttd14* ^*P75L*^ affects a conserved proline in the Walker A motif and abolished binding of TTD14 to GTP. This finding indicates that the P-loop domain indeed is the GTP binding site. It also suggests that GTP binding is important for TTD14 function as mutation of the site results in the observed *ttd14* phenotype.

A role of GTP binding proteins in the context of protein trafficking is well established. Important examples are the families of small GTPases, including Rho proteins that regulate actin organization, Rab proteins that regulate docking and fusion of vesicles at different organelles of the endocytic and secretory pathways, and Arf proteins that are involved in protein transport from Golgi to endoplasmic reticulum or at the trans-Golgi network [50]. Other examples for GTP binding proteins in this context are the microtubule forming subunits α and β tubulin and dynamin, which is required to pinch off clathrin-coated vesicles from membranes [51, 52]. Except for dynamin, GTP hydrolysis by these proteins is not used to generate force but rather to induce conformational changes in the GTP binding proteins that regulate protein function.

Besides to GTP, TTD14 also bound to the phospholipids PtdIns(3)P and phosphatidic acid (PA). Phosphoinositides that differ in the phosphorylation state of the inositol ring are distributed differentially in cellular compartments [44]. Accordingly, trafficking proteins that bind specific phosphoinositides are recruited to distinct membrane compartments [44]. PtdIns(3)P is located mainly in the early endosome [44]. Therefore, binding to PtdIns(3)P may recruit TTD14 to the early endosome in order to perform its function in TRPL recycling. PA is an intermediate in the biosynthesis of phosphoinositides and other phospholipids. In *Drosophila* photoreceptor cells, PA is generated during phototransduction that generates diacylglycerol (DAG) by phospholipase Cβ-mediated hydrolysis of phosphatidylinositol 4,5-bisphosphate. DAG in turn is translocated to the submicrovillar cisternae located at the base of the rhabdomere where it is phosphorylated to PA by the *rdgA*-encoded DAG kinase as a first step in the regeneration of rhabdomeral phosphatidylinositol 4,5-bisphosphate [53, 54]. Therefore, upon illumination, TTD14 could be recruited to the base of the rhabdomere via binding to PA, where it could assist light-triggered internalization of TRPL. However, an obvious light-dependent redistribution of myc-tagged TTD14 was not observed in immunocytochemical experiments (see Fig 3D). It has also been suggested that PA levels are critical for apical membrane transport events required for rhabdomere biogenesis in *Drosophila* photoreceptors as elevated PA levels in *Drosophila* mutants caused defects in rhabdomere biogenesis [55]. However, defective biogenesis of rhabdomeres during development was not observed in *ttd14* mutants and TRPL was properly transported to the rhabdomeres in young flies, arguing against a role of TTD14 in apical membrane trafficking events required for rhabdomere biogenesis.

As protein domain prediction did not reveal any lipid binding domain in the TTD14 protein, we performed a BLAST search with low stringency which revealed regions within TTD14 that showed amino acid sequence identities to vertebrate proteins between 30% and 37%. Homologous regions in vertebrate proteins that appeared in more than one species in the BLAST screen and contained specific protein domains within the homologous region included the Phox homologous- (PX) domain within vertebrate sorting nexin-8 homologs. This domain displayed sequence similarity with amino acids 254–320 of TTD14 (S1 Table). PX-domains were first identified in NADPH oxidase subunits, sorting nexins, and PtdIns(3)P-kinases [56]. These domains bind to phosphoinositides and sorting nexins are typically recruited to the early endosome membrane by binding to PtdIns(3)P via their PX-domain [57, 58]. Besides in the PX-domain there is little amino acid sequence conservation among the various sorting nexins and the PX-domain is located at varying positions. Sorting nexins are involved in the recycling of internalized proteins. They can be part of retromer complexes that sort internalized proteins in early or late endosomes away from the lysosomal degradation pathway and enable their trafficking back to the *trans*-Golgi network or to the plasma membrane [24, 59]. This function would be in accordance with a function of TTD14 in TRPL recycling. Yet, TTD14 is not an ortholog of a known sorting nexin as there is no significant sequence similarity over the whole protein length to any of the described 33 vertebrate sorting nexins. Given the weak sequence conservation between different classes of sorting nexins and the functional analogy of TTD14 with sorting nexins, it is tempting to speculate that TTD14 represents a new class of sorting nexin, composed of a GTP-binding domain and a putative PX-domain that is present in *Drosophila* and other invertebrates but not in vertebrates.

### Photoreceptor degeneration in the *ttd14* mutant

The *ttd14* ^*P75L*^ mutant displays a light-dependent late onset photoreceptor degeneration as ERG amplitudes diminished and rhabdomere structure became distorted when flies were kept in a 12 hours light / 12 hours dark cycle for more than 7 days (see Figs 6B, 6C and 7 and S4 Fig). While defects in the phototransduction cascade that result in constitutively open TRP channels, for example in *rdgA* or *trp* ^*P365*^ mutants, lead to fast photoreceptor degeneration within days after eclosion [54, 60, 61] light-dependent late onset photoreceptor degeneration of *Drosophila* photoreceptor cells can result from defective Rh1 transport [2, 62]. Accordingly, mutations in proteins required to transport Rh1 to the rhabdomere like XPORT, Rab11 or Crag cause photoreceptor degeneration [9, 18, 63]. Importantly, mutations in Vps26 or Vps35, components of the retromer complex, also cause light-dependent late onset photoreceptor degeneration [23]. This has been attributed to a failure in Rh1 recycling, which then accumulates in late endosomes or lysosomes and exerts a toxic effect on photoreceptor cells possibly due to an overload of the endolysosomal system [23]. Since *ttd14* mutants display a defect in TRPL recycling we wondered if they also have a defect in Rh1 recycling that would then cause photoreceptor degeneration. In contrast to TRPL, in flies dark-adapted for 28 d, Rh1 (and also TRP) were properly located in the rhabdomere as revealed by water immersion microscopy (Fig 5A). In addition, in the *ttd14* ^*P75L*^ mutant, over night illumination of flies with white light under conditions, which markedly reduced Rh1 levels in the *Vps26* ^*1*^ mutant [23], resulted in the same Rh1 levels as in wild type. Finally, while a reduction of the Rh1 content by genetic means or by vitamin A deprivation rescued photoreceptor cells from degeneration in the *Vps26* ^*1*^ mutant [23], Vitamin A deprivation did not rescue degeneration in the *ttd14* ^*P75L*^ mutant kept under a 12 hours light /12 hours dark cycle (see Figs 6E and 7 and S4 Fig). Thus, the defective *ttd14* allele does not seem to affect Rh1 recycling or trafficking of Rh1 to the rhabdomere, and photoreceptor degeneration caused by *ttd14* ^*P75L*^ cannot be attributed to defective Rh1 trafficking. These findings indicate that TTD14 is specifically required for TRPL recycling but not Rh1 recycling, either because TRPL utilizes a different recycling pathway than Rh1 or because TTD14 specifically recruits TRPL to a common recycling pathway.

What then might be the reason for photoreceptor degeneration in *ttd14* ^*P75L*^ flies? It can be excluded that the lack of TRPL in the rhabdomeres, observed in older *ttd14* ^*P75L*^ flies irrespective of the light condition, underlies photoreceptor degeneration as photoreceptors of the *trpl* ^*302*^ null mutant do not degenerate [46]. Rather than by Rh1 accumulation, degeneration of photoreceptor cells in *ttd14* ^*P75L*^ could be caused by accumulation of TRPL in the endocytic pathway. However, the amount of TRPL in photoreceptor cells is much smaller than the amount of Rh1, and dark-kept *ttd14* ^*P75L*^ flies, in which TRPL was found to be accumulated in the cell body, showed only little photoreceptor degeneration. Since a homozygous *ttd14* ^*P75L*^ mutant is lethal during the larval stage while all known *trpl* mutants are viable, it is likely that other membrane proteins require TTD14 for their recycling. Therefore, other proteins requiring TTD14 for recycling might be involved in photoreceptor degeneration. These proteins remain to be identified.

## Materials and Methods

### Fly stocks and genetics

The following strains of *Drosophila* were used: *yw*, *w* ^1118^ (here referred to as wild type), Oregon R (wild type with red eyes), *ninaE* ^*17*^ [64], *trp* ^*343*^ [65], *yw;;P[Rh1>TRPL-eGFP*,*y* ^*+*^ *]* [35], *yw;P[Rh1>Rh1-eGFP*,*w* ^*+*^ *]* [66], *yw;;P[Rh1>TRP-eGFP*,*y* ^*+*^ *]* [67], *yw*,*ey>flp; FRT42D*,*ttd14* ^*P75L*^ */CyO*, *yw; FRT42D*,*w* ^*+*^,*2R11*.*5(lth)/CyO* [39], *yw; FRT42D*,*w* ^*+*^,*2R11*.*5(lth)/CyO; P[Rh1>TRPL-eGFP*,*y* ^*+*^ *]*, *yw*,*P[Rh1>ttd14-A-myc*,*y* ^*+*^ *]*, *yw;;att86Fb[Rh1>ttd14-A*,*w* ^*+*^ *]*, *yw;;att86Fb[Rh1>ttd14-B*,*w* ^*+*^ *]*, *yw*, *ey>flp*; FRT42D,*ttd14* ^*KG03769[w+]*^ */CyO*, *yw*,*ey>flp; P[Rh1>Rh1-eGFP*,*w* ^*+*^ *]*, *FRT42D*,*ttd14* ^*P75L*^ */CyO*, *yw; FRT42D*,*ttd14* ^*P75L*^ */CyO*, *P[ActGFP*,*w* ^*+*^ *]* ^*JMR1*^, *yw; FRT42D*,*ttd14* ^*KG03769[w+]*^ */CyO*, *P[ActGFP*,*w* ^*+*^ *]* ^*JMR1*^.

Flies were raised on standard cornmeal food at 25°C unless indicated otherwise. Flies were either kept in the dark or in a 12 hours light / 12 hours dark cycle using white light illumination (1300 lux). For vitamin A deprivation, flies were raised and kept on food containing 10% dry yeast, 10% sucrose, 2% agar, and 0,02% cholesterol. For the analysis of TRPL ion channel translocation, flies were kept in the dark for the indicated period and were then illuminated with orange light (acrylic glass cut off filter transmitting light >560 nm, ~200 lux) for 16 hours. Light-raised flies were dissected under white light whereas dark-raised flies were dissected under dim red light (Schott RG 630, cold light source KL1500, Schott). For the analysis of the amount of Rh1 protein flies were illuminated with white light (1800 lux). To map the mutation *in ttd14*, *yw*,*ey>flp*, *P[Rh1>TRPL-eGFP*,*y* ^*+*^ *]*; FRT42D,*ttd14* ^*P75L*^/CyO mutant flies were crossed to the Bloomington 2R Deficiency Kit and the offspring was screened for lethality and TRPL translocation. In addition, the deficiency stocks Df(2R)ED3610 (54F1-55C8), Df(2R)BSC483 (55A1-55B7), Df(2R)334 (55B2-55C4), Df(2R)3636 (55B8-55E3), Df(2R)Excel7153 (55B9-55C1), Df(2R)BSC337 (55B11-55C9), Df(2R)ED3683 (55C2-56C4), Df(2R)BSC335 (55C6-55F1), Df(2R)BSC399 (55D1-55E10), Df(2R)BSC339 (55E2-55F6), and Df(2R)Excel7158 (55E9-55F6) were used to refine the region of the lethal *ttd14* ^*P75L*^ mutation.

For the analysis of TRPL-eGFP localization in the eye of *ttd14* ^*P75L*^ mutant *Drosophila*, mosaic eyes were generated by crossing *yw*,*ey>flp; FRT42D*,*ttd14* ^*P75L*^ */CyO* females with *yw; FRT42D*,*w* ^*+*^,*2R11*.*5(lth)/CyO; P[Rh1>TRPL-eGFP*,*y* ^*+*^ *]* males. Female offspring with mosaic eyes (*yw/yw*,*ey>flp; FRT42D*,*ttd14* ^*P75L*^ */FRT42D*,*w* ^*+*^,*2R11*.*5(lth); P[Rh1>TRPL-eGFP*,*y* ^*+*^ *]/+*) was analyzed for the localization of TRPL-eGFP using the deep pseudopupil and water immersion microscopy. For wild type flies, *yw* females were crossed with *yw;;P[Rh1>TRPL-eGFP*,*y* ^*+*^ *]* males. Female offspring of the genotype *yw;;P[Rh1>TRPL-eGFP*,*y* ^*+*^ *]/+* was analyzed. To analyze the rescue construct (*Rh1>ttd14-A-myc*) in *ttd14* ^*P75L*^ mutant mosaic clones, we crossed females expressing the rescue construct on the X-chromosome (*yw*,*P[Rh1>ttd14-A-myc*,*y* ^*+*^ *]*) with *yw; FRT42D*,*w* ^*+*^,*2R11*.*5(lth)/CyO; P[Rh1>TRPL-eGFP*,*y* ^*+*^ *]* males. F-1 males without the *CyO* balancer (*yw*,*P[Rh1>ttd14-A-myc*,*y* ^*+*^ *]; FRT42D*,*w* ^*+*^,*2R11*.*5(lth)/+; P[Rh1>TRPL-eGFP*,*y* ^*+*^ *]/+*) were then crossed with *yw*,*ey>flp; FRT42D*,*ttd14* ^*P75L*^ */CyO* females. In the F-2 generation, females carrying mosaic eyes were selected for the presence of the TRPL-eGFP reporter by fluorescence microscopy using a Leica MZ16 F stereomicroscope prior to water immersion microscopy. Analyzed flies had the following genotype: *yw*,*P[Rh1>ttd14-A-myc*,*y* ^*+*^ *]/yw*,*ey>flp; FRT42D*,*ttd14* ^*P75L*^ */FRT42D*,*w* ^*+*^, *2R11*.*5(lth); P[Rh1>TRPL-eGFP*,*y* ^*+*^ *]/+*.

For the analysis of the rescue by the non-tagged *Rh1>ttd14-A* and *Rh1>ttd14-B* construct, the transgenic males *yw;;att86Fb[Rh1>ttd14-A*,*w* ^*+*^ *]* and *yw;;att86Fb[Rh1>ttd14-B*,*w* ^*+*^ *]* were crossed to *yw*,*ey>flp; FRT42D*,*ttd14* ^*P75L*^ */CyO* females. The heterozygous rescue construct can be traced by the orange eye color. F-1 males with orange colored eyes and without the *CyO* balancer (*yw*,*ey>flp; FRT42D*,*ttd14* ^*P75L*^ */+; att86Fb[Rh1>ttd14-A or B*,*w* ^*+*^ *]*/+) were then crossed with *yw; FRT42D*,*w* ^*+*^,*2R11*.*5(lth)/CyO; P[Rh1>TRPL-eGFP*,*y* ^*+*^ *]* females. In the F-2 generation, females carrying mosaic eyes composed of orange and red clones (*yw/yw*,*ey>flp; FRT42D*,*ttd14* ^*P75L*^ */FRT42D*,*w* ^*+*^,*2R11*.*5(lth); P[Rh1>TRPL-eGFP*,*y* ^*+*^ *]/att86Fb [Rh1>ttd14-A or B*,*w* ^*+*^ *]*) were analyzed.

For the analysis of the TRPL localization in the *ttd14* ^KG03769[w+]^ allele, this mutant allele was recombined with the *FRT42D* locus. Next, *yw*, *ey>flp; FRT42D*,*ttd14* ^*KG03769[w+]*^ */CyO* males were crossed with *yw; FRT42D*,*w* ^*+*^,*2R11*.*5(lth)/CyO; P[Rh1>TRPL-eGFP*,*y* ^*+*^ *]* females. In the F-1 generation, females lacking the *CyO* balancer (*yw/yw*,*ey>flp; FRT42D*,*ttd14* ^*KG03769[w+]*^ */FRT42D*,*w* ^*+*^,*2R11*.*5(lth); P[Rh1>TRPL-eGFP*,*y* ^*+*^ *]/+*) were analyzed. For the wild type control *yw;;P[Rh1>TRPL-eGFP*,*y* ^*+*^ *]* flies were crossed with Oregon R flies and F-1 females (*yw/+;; P[Rh1>TRPL-eGFP*,*y* ^*+*^ *]/+*) were analyzed.

For the analysis of TRP-eGFP localization, flies carrying the TRP-eGFP reporter (*yw;;P[Rh1>TRP-eGFP*,*y* ^*+*^ *]*) were crossed with *yw*,*ey>flp; FRT42D*,*ttd14* ^*P75L*^ */CyO* females. F-1 males lacking the *CyO* balancer (*yw*,*ey>flp; FRT42D*,*ttd14* ^*P75L*^ */+; P[Rh1>TRP-eGFP*,*y* ^*+*^ *]/+*) were then crossed to *yw; FRT42D*,*w* ^*+*^,*2R11*.*5(lth)/CyO* females. In the F-2 generation female flies with mosaic eyes were selected for the presence of the TRP-eGFP reporter using a Leica MZ16 F stereomicroscope prior to water immersion microscopy. Analyzed flies had the following genotype: *yw/yw*,*ey>flp; FRT42D*,*ttd14* ^*P75L*^ */FRT42D*,*w* ^*+*^,*2R11*.*5(lth); P[Rh1>TRP-eGFP*,*y* ^*+*^ *]/+*. For the wild type control, *yw* females were crossed to *yw;;P[Rh1>TRP-eGFP*,*y* ^*+*^ *]* males and F-1 females (*yw;;P[Rh1>TRP-eGFP*,*y* ^*+*^ *]/+*) were subjected to water immersion microscopy.

For the analysis of Rh1-eGFP localization in water immersion microscopy and immunocytochemistry, the *P[Rh1>Rh1-eGFP*,*w* ^*+*^ *]* construct was recombined to the *FRT42D*,*ttd14* ^*P75L*^ chromosome resulting in the fly stock *yw*,*ey>flp; P[Rh1>Rh1-eGFP*,*w* ^*+*^ *]*, *FRT42D*,*ttd14* ^*P75L*^ */CyO*. Flies from this stock were crossed with *yw; FRT42D*,*w* ^*+*^,*2R11*.*5(lth)/CyO* flies and F-1 females with mosaic eyes were analyzed (*yw/yw*,*ey>flp; P[Rh1>Rh1-eGFP*,*w* ^*+*^ *]*, *FRT42D*,*ttd14* ^*P75L*^ */FRT42D*,*w* ^*+*^,*2R11*.*5(lth)*). The *w* ^*+*^ marker of the heterozygous *Rh1>Rh1-eGFP* construct results in a faint orange eye color while flies homozygous for the *Rh1>Rh1-eGFP* construct exhibit dark orange colored eyes. As the F-1 females with mosaic eye clones display a faint orange eye color, we assume that the *Rh1>Rh1-eGFP* construct is present with one copy in this genetic background. We conclude that the *Rh1>Rh1-eGFP* construct is localized on the left arm of the second chromosome which is not affected by FRT-mediated mitotic recombination. Therefore, for the wild type control, *yw* females were crossed to *yw;P[Rh1>Rh1-eGFP*,*w* ^*+*^ *]* males and F-1 females (*yw;P[Rh1>Rh1-eGFP*,*w* ^*+*^ *]/+*) were subjected to water immersion microscopy.

For immunoblot, immunocytochemistry and ERG experiments, *yw*,*ey>flp; FRT42D*,*ttd14* ^*P75L*^ */CyO* flies were crossed with *yw; FRT42D*,*w* ^*+*^,*2R11*.*5(lth)/CyO* flies and female F-1 flies with mosaic eyes (*yw/yw*,*ey>flp; FRT42D*,*ttd14* ^*P75L*^ */FRT42D*,*w* ^*+*^,*2R11*.*5(lth)*) were used for analysis. *w* ^1118^ was used as wild type control. To analyze the rescue construct (Rh1>*ttd14-A*-myc) in *ttd14* ^*P75L*^mutant mosaic clones, we crossed females expressing the rescue construct on the X-chromosome (*yw*,*P[Rh1>ttd14-A-myc*,*y* ^*+*^ *]*) with *yw; FRT42D*,*w* ^*+*^,*2R11*.*5(lth)/CyO* males. F-1 males without the *CyO* balancer (*yw*,*P[Rh1>ttd14-A-myc*,*y* ^*+*^ *]; FRT42D*,*w* ^*+*^,*2R11*.*5(lth)/+*) were then crossed with *yw*,*ey>flp; FRT42D*,*ttd14* ^*P75L*^ */CyO* females. In the F-2 generation, females carrying mosaic eyes (*yw*,*P[Rh1>ttd14-A-myc*,*y* ^*+*^ *]/yw*,*ey>flp; FRT42D*,*ttd14* ^*P75L*^ */FRT42D*,*w* ^*+*^,*2R11*.*5(lth)*) were analyzed.

For the analysis of the subcellular localization of the TTD14 protein by immunocytochemistry *yw*,*P[Rh1>ttd14-A-myc*,*y* ^*+*^ *]* flies were crossed with *yw;;P[Rh1>TRPL-eGFP*,*y* ^*+*^ *]* flies and female F-1 flies, carrying both reporter constructs (*yw*,*P[Rh1>ttd14-A-myc*,*y* ^*+*^ *]/+;;P[Rh1>TRPL-eGFP*,*y* ^*+*^ *]/+*) were analyzed.

To address the larval lethality of mutant alleles of the *ttd14* gene, the mutant alleles were crossed with a GFP-labeled *CyO*-Balancer (*yw; FRT42D*,*ttd14* ^*P75L*^ */CyO*, *P[ActGFP*,*w* ^*+*^ *]* ^*JMR*^ and *yw; FRT42D*,*ttd14* ^*KG03769[w+]*^ */CyO*, *P[ActGFP*,*w* ^*+*^ *]* ^*JMR1*^ respectively) and development of non-fluorescent homozygous mutant F-1 larvae was traced.

### Generation of rescue constructs and transgenic flies

For the generation of the Rh1>*ttd14-A* and Rh1>*ttd14-B* rescue constructs, the entire coding sequence, but not the 5´ and 3´ untranslated region of *ttd14-A* and *ttd14-B* was PCR amplified from cDNA derived from fly heads. Restriction sites were introduced in the primer pair 5´-TTCCCGAATTCGAAGACATG-3 (EcoRI) and 5´-GAGTCAACATAATCGATAGCCA-3´ (ClaI) for the amplification of *ttd14-A* and 5´-TTCCCGAATTCGAAGACATG-3´ (EcoRI) and 5´-GTCCCTGAATCGATTTTGCACAC-3´ (ClaI) for the amplification of *ttd14-B*. PCR fragments were cloned into a modified pBluescript II SK vector (Stratagene) between the Rh1 minimal promoter (base pairs -833 to +67) and the last 0.6 kb of the Rh1 3´ untranslated region using the EcoRI and ClaI restriction sites. After a KpnI site in the Rh1 promoter was eliminated with a site directed mutagenesis Kit (Agilent) using the mutagenesis primer 5´-CAGAATCCAGGAACCCTGAGTACCGGATCC-3´, the *Rh1>ttd14-A*(*B*) construct was excised from the pBluescript vector using a NotI/KpnI digest and cloned into the pattB vector [68]. The final pattB *Rh1*>*ttd14*-*A* and -*B* clones were verified by DNA sequencing (Qiagen). Transgenic flies were generated using site specific recombination by injecting the pattB *Rh1*>*ttd14-A*/*B* vector (200 ng/μl) into y^1^ M{vas-int.Dm}ZH-2A w*; M{3xP3-RFP.attP}ZH-86Fb embryos. For the generation of the *Rh1*>*ttd14-A*-*myc* rescue construct, a PCR amplified *ttd14-A* construct encoding amino acids 1 to 472 of TTD14-A and a C-terminal myc-tag obtained from the vector pSF-CMV-COOH-EKT-CMyc2 (Oxford Genetics) was cloned in the pENTR 1A vector (Invitrogen). Using the Gateway system (Invitrogen), the *ttd14-A*-*myc* sequence was recombined with a modified pYC4 vector containing a DEST cassette between the Rh1 minimal promoter (base pairs -833 to +67) and the last 0.6 kb of the 3´untranslated region of Rh1 [67]. The final pYC4 *Rh1>ttd14-A-myc* clone was verified by DNA sequencing (GATC Biotech). The construct was injected into *yw* embryos.

### Expression and purification of recombinant TTD14 protein

Full length *ttd14-A* was PCR amplified from cDNA derived from fly heads and cloned in the pQE30 vector (Qiagen), which encodes a N-terminal His-tag, using BamHI and SalI sites. For generating a construct encoding the mutated TTD14[P75L] protein, the respective codon was mutated using the Quick Change Lightning Site-Directed Mutagenesis Kit (Agilent) as described in the instruction manual. The resulting pQE30 *ttd14-A* and pQE30 *ttd14-A[P75L]* clones were verified by DNA sequencing (Qiagen) and transformed in *E*. *coli* M15 cells (Qiagen). For the purification of native TTD14 or TTD14[P75L] protein, expression of the recombinant protein was induced by 1 mM IPTG for 2 hours at 30°C. The pellet of 1 liter bacteria culture was lysed in 20 ml lysis buffer (50 mM Tris, pH 8.0, 300 mM NaCl, 10 mM Imidazol, 1% Triton X-100, 2 mM DTT, 50 μM APMSF) using a French press (OneShot; Constant Systems) and incubated with 500 μl Ni-NTA agarose (Qiagen) for 60 min at 4°C. After three wash steps in 20 ml wash buffer each (50 mM Tris/HCl, pH 8.0, 300 mM NaCl, 50 mM Imidazol, 50 μM APMSF), the TTD14 protein was eluted from the Ni-NTA agarose with 400 μl elution buffer (50 mM Tris, pH 8.0, 300 mM NaCl, 250 mM Imidazol, 50 μM APMSF). For the purification of TTD14 protein under denaturating conditions, expression of the recombinant protein was induced by 1 mM IPTG for 2 hours at 37°C. The pellet of a 300 ml bacteria culture was homogenized in 10 ml lysis buffer (8 M Urea, 100 mM Na_2_HPO_4_,/NaH_2_PO_4_, pH 8.0, 10 mM Tris/HCl pH 8.0, 20 mM β-mercaptoethanol, 10 mM Imidazol) and incubated with 2 ml of Ni-NTA agarose (Qiagen) for 30 min at room temperature. After three wash steps in 20 ml wash buffer each (8 M Urea, 100 mM Na_2_HPO_4_,/NaH_2_PO_4_, pH 6.3, 10 mM Tris/HCl pH 6.3, 10 mM Imidazol), the TTD14 protein was eluted with 1 ml elution buffer (50 mM Tris/HCl, pH 7.5, 300 mM NaCl, 250 mM Imidazol).

### Generation of an anti-TTD14 antibody

1 μg of TTD14 protein purified under denaturating conditions was used to immunize two rabbits for 61 days (Pineda Germany). IgG were purified from the serum using a HiTrap Protein A HP-column (GE Healthcare) according to the manufacturer’s instructions.

### Phospholipid binding assay

PIP-Strips (Echelon Research Laboratories) which contain various phospholipids at distinct spots (100 pmol) were blocked in TBS-T (10 mM Tris/HCl pH 8.0; 150 mM NaCl, 0.1% Tween-20) containing 3% bovine serum albumin (BSA). The membrane was incubated with recombinant TTD14 protein purified under native conditions (0.4 μg/ml) in TBS-T plus 3% BSA for 90 min at 4°C. After three wash steps for 5 min each in TBS-T, the membrane was incubated with the α-TTD14 antibody in TBS-T plus 3% BSA for 90 min at 4°C, washed again three times for 5 min each in TBS-T and finally incubated with horseradish peroxidase-coupled anti-rabbit IgG (1:10,000 Sigma) in TBS-T plus 3% BSA for 90 min at 4°C. Lipid-bound protein was detected by enhanced chemiluminescence (0.091 M Tris-HCl pH 8.6; 0.0227% (w/v) luminol; 0.01% (w/v) para-hydroxycoumarin acid 0.01% H_2_O_2_) using a ChemiDocXRS^+^ Imaging system (Bio-Rad).

### GTP binding assay

20 μg of recombinant wild type TTD14 or TTD14[P75L] protein was diluted to 225 μl with binding buffer (20 mM Tris-HCl pH 7.0; 150 mM NaCl; 5 mM MgCl_2_; 0.1% (v/v) Triton X-100) and incubated with agitation (1000 rpm) at 4°C for 1 hour with 25 μl of either control agarose (Pierce), ATP-agarose (Sigma Aldrich) or GTP-agarose (Sigma Aldrich) pre-equilibrated in binding buffer. Beads were centrifuged at 4°C at 13,000 g for 2 min and washed three times with 1 ml ice-chilled binding buffer. Proteins were eluted by adding 50 μl of binding buffer containing either 25 mM ATP or 25 mM GTP for 5 min on ice. After centrifugation (2 min at 13,000g at 4°C) 15 μl of the resulting supernatant was subjected to immunoblot analysis.

### Preparation of proteins for immunoblot analysis

For immunoblot analyses of TRPL, TRP, Rh1, and Tubulin in wild type and *ttd14* ^*P75L*^ mutant flies (Figs 4D, 5C and 6D and S3 Fig), fly heads were homogenized in SDS extraction buffer (4% SDS, 1 mM EDTA, 75 mM Tris/HCl, pH 6.8) using 4 μl of extraction buffer per head and incubated for 1 hour at room temperature. After 10 min centrifugation at 16,000 g to remove debris the supernatant was subjected to SDS-PAGE. For immunoblot analysis of TTD14 protein in wild type and *ttd14* mutant flies (Fig 3B), eye cups were dissected and homogenized in Tris-buffer (50 mM Tris/HCl pH 8.0; 150 mM NaCl; 50 μM APMSF; 1 μl per eye) and extraction was carried out for 30 min on ice. The supernatant obtained after 10 min centrifugation at 16,000 g was subjected to SDS-PAGE. For immunoblot analysis of membrane and soluble proteins (Fig 3C), fly heads were homogenized in Tris-buffer (50 mM Tris/HCl pH 8,0; 150 mM NaCl; 50 μM APMSF; 2 μl per head) and extraction was carried out for 30 min at room temperature. A 3 min centrifugation step at 2,500 g was applied to remove cuticular particles of the fly head. The supernatant was subjected to ultracentrifugation (10 min at 100,000 g, 4°C) and the resulting supernatant was loaded as soluble fraction on a SDS-gel. The membrane pellet was washed three times in Tris-buffer and solubilized in SDS extraction buffer (4% SDS, 1 mM EDTA, 75 mM Tris/HCl, pH 6.8; 1 μl per head) for 20 min at room temperature and loaded as membrane fraction on a SDS-gel.

### Immunoblot analysis

SDS-PAGE was performed according to Laemmli [69] using 10% or 12% polyacrylamide gels. Separated proteins were electrophoretically transferred to polyvinylidene difluoride membranes (Bio-Rad). The membrane was then blocked for 20 min in TBS-T with 5% skim milk (10 mM Tris/HCl, pH 7.5, 150 mM NaCl, 0.1% Tween 20, 5% skim milk). α-TRPL [34], α-TRP (Mab83F6; Developmental Studies Hybridoma Bank, University of Iowa), α-Rh1 (4C5; Developmental Studies Hybridoma Bank), α-tubulin (E7; Developmental Studies Hybridoma Bank, University of Iowa) and α-TTD14 antibodies were used for immunological detection in TBS-T with 5% skim milk over night at 4°C. Signals were detected by enhanced chemiluminescence ((0.091 M Tris-HCl pH 8.6; 0.0227% (w/v) luminol; 0.01% (w/v) para-hydroxycoumarin acid; 0.01% H_2_O_2_) using the ChemiDocXRS^+^ Imaging system (Bio-Rad). Quantification of immunoblot signals was performed with Image Lab 4.0 (Bio-Rad) by determining the integrated density of each protein band. The Rh1 signals were normalized by the TRP signals of the same sample.

### Fluorescence microscopy

Fluorescence in the deep pseudopupil was observed in CO_2_-anaesthetized flies using a Leica MZ16 F stereomicroscope with 63x magnification and the GFP3 filter set. Images were captured with a Leica DFC420 C camera. Water immersion microscopy of eGFP-tagged proteins in intact eyes was performed as previously described [35]. Living flies were anaesthetized on ice, spiked on an insect needle, mounted with plasticine on an object slide and covered with ice-chilled distilled water. The eGFP fluorescence was observed with an AxioImager.Z1m microscope (Zeiss, Germany; objective: Achroplan 20X/0.5 water immersion). Images were captured with the AxioCamMrM (Zeiss) camera and the Axio-Vision 4.6/4.8 or Zen 2012 (Zeiss) software. For images of flies with white and orange colored eyes, exposure time was determined individually for every single eye to be closely below overexposure. For the analysis of the red colored wild type and *ttd14* ^*KG03769*^ eyes, an exposure time resulting in images closely below overexposure in 1 day old dark-adapted wild type flies was determined and applied for all other experimental conditions. For quantitative analyses of the relative TRPL-eGFP fluorescence in the rhabdomeres, fluorescence images obtained with the water immersion technique were analyzed with ImageJ 1.42j software (National Institute of Health, USA). The relative amount of TRPL-eGFP present in the rhabdomeres (R) was calculated using the formula *R* = (*I*r–*I*b)/[(*I*r–*I*b)+(*I*c–*I*b)], where *I*r, *I*b, and *I*c are the fluorescence intensities in the rhabdomeres, in the background, and in the cell body, respectively. Background intensities were determined in the center of the ommatidium where the rhabdomere of the R7/R8 cells is located. For each eye, three ommatidia were analyzed and five individual flies were analyzed per data point. The data were normalized to the value obtained for 1 day old dark raised wild type flies that was set to 100%. For determining the time course of rhabdomere degeneration flies expressing TRP-eGFP in R1-6 photoreceptors were inspected for the presence of R1-6 rhabdomeres using water immersion microscopy. Clearly visible rhabdomeres were scored 2, weakly visible rhabdomeres were scored 1 and absent rhabdomeres were scored 0. Three ommatidia per eye were scored, resulting in a degeneration score of 36 for fully intact eyes or in a score of 0 for fully degenerated eyes. The maximal degeneration score of 36 was set to 100%. Five individual flies were analyzed per data point.

### Immunocytochemistry

For immunocytochemical analyses, *Drosophila* eyes were fixed in 2% paraformaldehyde (PFA) in PBS (175 mM NaCl, 8 mM Na_2_HPO_4_, and 1.8 mM NaH_2_PO_4_, pH 7.2) for 1 hour at room temperature, and then washed twice in 0.1 M phosphate buffer (0.1 M Na_2_HPO_4_ and 0.1 M NaH_2_PO_4_, pH 7.2). Subsequently, three wash steps in 10% sucrose and two wash steps in 25% sucrose were performed for 15 min each. Eyes were finally infiltrated with 50% sucrose overnight at 4°C, cryofixed in melting pentane, and sectioned at 10 μm thickness in a Leica CM3050S cryostat (Leica, Germany) at −25°C. Cryosections were first fixed in 2% PFA in PBS for 10 min and then washed twice in PBS. After blocking of sections in PBS-T (1% BSA, 0.3% Triton X-100 in PBS) for 2 hours at room temperature, sections were incubated with α-TRPL [35], α-TRP (Mab83F6; Developmental Studies Hybridoma Bank of the University of Iowa) and α-myc (Sigma) in PBS-T overnight at 4°C. The sections were subsequently washed three times in PBS and were then incubated either with α-mouse-Alexa Fluor 488 and α-rabbit-Alexa Fluor 680 or with α-mouse-Alexa Fluor 680 and α-rabbit-Alexa Fluor 488 (Life Technololgies) in 0.5% fish gelatine and 0.1% ovalbumin in PBS for at least 4 hours at room temperature. Phalloidin-Alexa Fluor 546 (Life Technologies) was added to stain F-actin in rhabdomeres. The eGFP-tagged Rh1 protein was visualized by its own fluorescence. The sections were finally mounted in Mowiol 4.88 (Polyscience) and examined with an AxioImager.Z1m microscope (objective: EC Plan-Neofluar 40×/1.3 Oil, Zeiss, Germany) using the ApoTome module (Zeiss, Germany) at room temperature. Images were captured with the AxioCamMrM (Zeiss) camera and the Axio-Vision 4.6/4.8 or Zen 2012 (Zeiss) software.

### Electroretinogram measurements

For electroretinogram recordings, flies were immobilized in a pipette tip and mounted with a mixture of colophonium and bee’s wax (1:3). Electroretinogram recordings were performed at room temperature after 3 minutes of dark adaptation prior to the first orange light-stimulus. Light-stimuli of 5 s duration were delivered by an orange light-emitting diode (Roithner, Austria) and a blue light-emitting diode (Roithner, Austria) in a setup of two collimating lenses (Linos, Germany) within the light path. The light intensity at the position of the fly eye was 2.15 mW/cm^2^ for orange light and 1.3 mW/cm^2^ for blue light. A DPA-2FS amplifier (NPI electronic, Germany) with a low pass filter (700 Hz) was used for signal amplification. Analog-to-digital conversion was accomplished with a BNC-2090A rack-mounted terminal block (National Instruments, Germany) and a PCI-6221 PC card (National Instruments, Germany). Data recording was achieved by the Whole Cell Analysis Program software WinWCP 4.7.6. (University of Strathclyde). The recording electrode glass capillary was filled with Davenport solution (100 mM NaCl, 2 mM KCl, 1 mM CaCl_2_, 1.8 mM NaHCO_3_, pH 7.2).

### Electron microscopy

Fly heads were separated from the body, dissected into two halves and incubated in fixative solution (4% paraformaldehyde, 2.5% glutaraldehyde in 1 x PBS, pH 7.4) for 1 h at room temperature. The semi-heads were washed 3 times with 0.1 M sodium cacodylate buffer (pH 7.4) for 10 min and postfixed in 2% OsO_4_ in 0.1 M cacodylate buffer (pH 7.4) for 1 h. After 3 washes in 0.1 M sodium cacodylate buffer for 10 min the semi-heads were dehydrated through a graded series of ethanol from 30% to 100%. The dehydrated semi-heads were incubated in 100% propylene oxide twice for 10 min each, and then transferred to 50% propylene oxide: 50% Renlam M-1 resin (Serva Electrophoresis, Heidelberg, Germany) and incubated overnight. After that the semi-heads were incubated in 100% Renlam® M-1 resin overnight, embedded in 100% Renlam® M-1 resin and polymerized at 60°C for two days. Ultrathin sections (60–70 nm) were obtained using a Reichert Ultracut E microtome (Leica). Sections were counterstained with heavy metal staining (2% uranyl acetate in 50% ethanol; aq. 2% lead citrate). Ultrathin sections were analyzed in a Tecnai 12 BioTwin transmission electron microscope (FEI, Eindhoven, The Netherlands. Images were obtained with a charge-coupled device SIS Megaview3 SCCD camera (Surface Imaging Systems, Herzogenrath, Germany). Image contrast was adjusted with Adobe Photoshop CS using different tools.

## Supporting Information

S1 Fig TRPL trafficking in *ttd14* ^*P75L*^ mutants expressing rescue constructs and in red-eyed *ttd14* ^*KG03769*^ flies. Flies were kept in the dark for 1 day or for 7 days as indicated, illuminated with orange light for 16 hours and then again kept in darkness for 24 hours. (A) Water immersion microscopy detecting TRPL-GFP in *ttd14* ^*P75L*^ mutant eye clones expressing the rescue constructs *Rh1>ttd14*-*A* (left panel) or *Rh1>ttd14*-*B* (right panel). Flies expressing the rescue constructs had homozygous mutant orange colored eye clones, from which the images were obtained, and red heterozygous eye clones. The light-dependent translocation of TRPL-eGFP in these mutants resembles TRPL-eGFP translocation in wild type flies. Scale bar: 10 μm. (B) Water immersion microscopy detecting TRPL-GFP in eyes from red-eyed wild type flies (left panel) and in mutant eye clones of the *ttd14* ^*KG03769*^ mutant (right panel). Eyes of the *ttd14* ^*KG03769*^ mutant were red such that homozygous mutant eye clones could not be distinguishes from heterozygous eye clones in *ttd14* ^*KG03769*^. However, typically 80–90% homozygous mutant eye clones are formed by the FRT/FLP system used here. Differences in TRPL-eGFP fluorescence between wild type and *ttd14* ^*KG03769*^ mutant, that correspond to the phenotypes observed in white eye clones of *ttd14* ^*P75L*^ (see Fig 3), were observed in light-adapted 1 d old flies (compare b,h), 1 d old flies after the second dark-adaptation (compare c,i) and dark-adapted 7 d old flies (compare d,j and f,l) in most of the observed eyes, when images were captured with exactly the same exposure time.
(TIF)

S2 Fig TRPL localization after dark-adaptation in *ttd14* ^*P75L*^ mutants in comparison to phosphorylation deficient TRPL. Upper row: Localization of native TRPL on cross sections through ommatidia from *ttd14* ^*P75L*^ mutant eye clones. Flies were aged for 7 days in darkness and subsequently illuminated for 16 hours with orange light and subjected to a second dark-adaptation for another 24 hours. Cross sections were probed with an anti-TRPL antibody (green, left column) and Alexa Fluor 546-coupled phalloidin (red, middle column). Merged panels are shown in the right column. Lower row: Localization of phosphorylation deficient TRPL8x-eGFP protein on cross sections through ommatidia derived from flies expressing the TRPL8x-eGFP construct under the control of the Rh1 promoter in a *trpl* ^*302*^ mutant background. Flies were aged for 3–5 days in darkness and, illuminated for 16 hours with orange light and again dark-adapted for another 24 hours. TRPL8x-eGFP was visualized by its GFP fluorescence (green, left column). Rhabdomeres were stained with Alexa Fluor 546-coupled phalloidin (red, middle column). Merged panels are shown in the right column. TRPL8x-eGFP fluorescence in the cell body appears in distinct spots (arrowheads). Scale bar: 5 μm.
(TIF)

S3 Fig Protein abundance of Rh1 and TRP is not affected in the *ttd14* ^*P75L*^ mutant. (A) Immunoblot analysis of TRP, Rh1 and Tubulin from wild type heads or from heads with *ttd14* ^*P75L*^ mutant eye clones (equivalent of 3 heads per lane) (same blot as in Fig 4D). Freshly eclosed flies (1 day) or flies kept in the dark for 7 days were analyzed immediately (first black bars) or subjected to orange light illumination for 16 hours (white bars) followed by 24 hours of darkness (second black bars). The blots were probed with α-TRP, α-Rh1 and α-Tubulin antibodies as indicated. The size of molecular weight markers in kilo Dalton is shown at the left. (B,C) Quantification of the TRP (B) and Rh1 (C) levels normalized to Tubulin. The TRP and Rh1 levels of 1 day old flies illuminated for 16 hours (second column) was set to 100% each. Error bars show SEM (n = 5). No significant differences in the amount of TRP and Rh1 could be detected between wild type and *ttd14* ^*P75L*^ mutant flies.
(TIF)

S4 Fig Water immersion microscopy images of TRP-eGFP fluorescence in eyes of wild type flies or *ttd14* ^*P75L*^ mutant eye clones. Flies were aged for the indicated number of days in a 12 hours light / 12 hours dark cycle and kept either on regular food (A) or on a vitamin A-deprived diet (low vitA) (B). Progressive loss of rhabdomeres and the regular rhabdomeral structure was observed in the *ttd14* ^*P75L*^ mutant eye clones both on regular and vitamin-deprived food, but not in wild type eyes. Scale bar: 10 μm.
(TIF)

S1 Table Domains in vertebrate proteins that are homologous to *Drosophila* TTD14. (DOCX)
