# Supplementary figures and images for: The GTP- and Phospholipid-Binding Protein TTD14 Regulates Trafficking of the TRPL Ion Channel in Drosophila Photoreceptor Cells
Source: PLoS Genet. 2015 Oct 28;11(10):e1005578. doi: 10.1371/journal.pgen.1005578 (PMC4624897; doi:10.1371/journal.pgen.1005578)

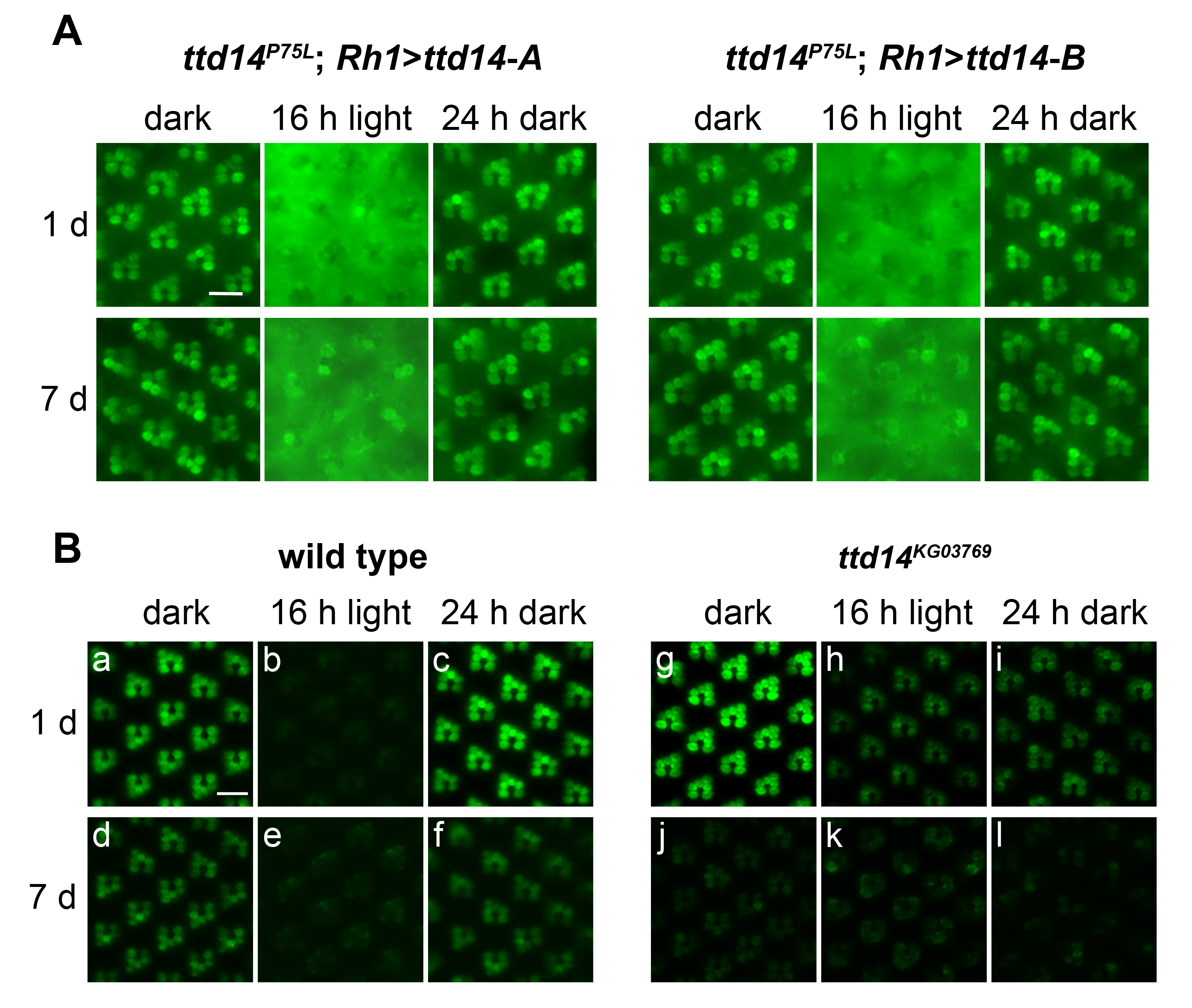

Supplement: S1 Fig — Flies were kept in the dark for 1 day or for 7 days as indicated, illuminated with orange light for 16 hours and then again kept in darkness for 24 hours. (A) Water immersion microscopy detecting TRPL-GFP in ttd14 P75L mutant eye clones expressing the rescue constructs Rh1>ttd14-A (left panel) or Rh1>ttd14-B (right panel). Flies expressing the rescue constructs had homozygous mutant orange colored eye clones, from which the images were obtained, and red heterozygous eye clones. The light-dependent translocation of TRPL-eGFP in these mutants resembles TRPL-eGFP translocation in wild type flies. Scale bar: 10 μm. (B) Water immersion microscopy detecting TRPL-GFP in eyes from red-eyed wild type flies (left panel) and in mutant eye clones of the ttd14 KG03769 mutant (right panel). Eyes of the ttd14 KG03769 mutant were red such that homozygous mutant eye clones could not be distinguishes from heterozygous eye clones in ttd14 KG03769. However, typically 80–90% homozygous mutant eye clones are formed by the FRT/FLP system used here. Differences in TRPL-eGFP fluorescence between wild type and ttd14 KG03769 mutant, that correspond to the phenotypes observed in white eye clones of ttd14 P75L (see Fig 3), were observed in light-adapted 1 d old flies (compare b,h), 1 d old flies after the second dark-adaptation (compare c,i) and dark-adapted 7 d old flies (compare d,j and f,l) in most of the observed eyes, when images were captured with exactly the same exposure time. (TIF) [file pgen.1005578.s001.tif]

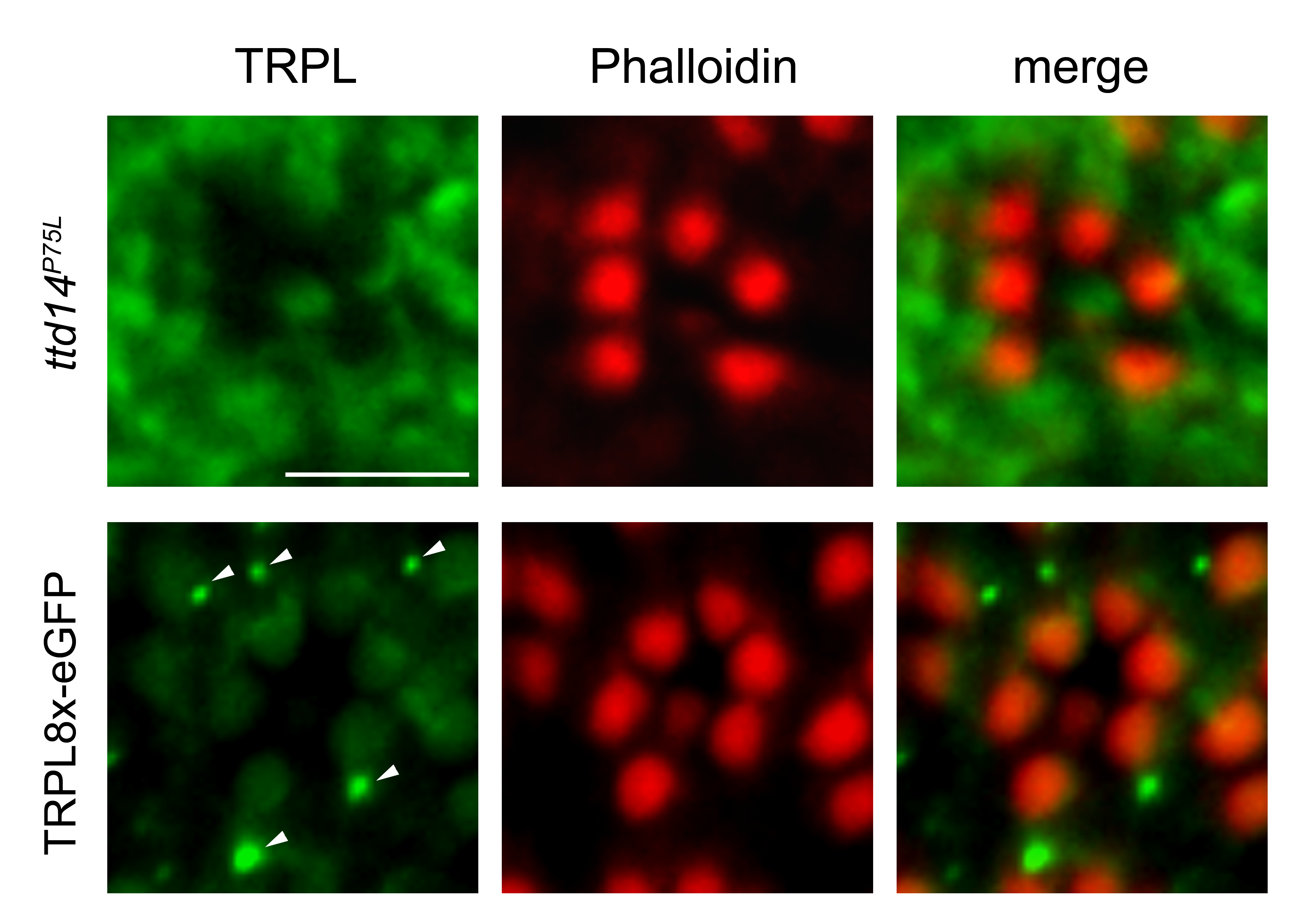

Supplement: S2 Fig — Upper row: Localization of native TRPL on cross sections through ommatidia from ttd14 P75L mutant eye clones. Flies were aged for 7 days in darkness and subsequently illuminated for 16 hours with orange light and subjected to a second dark-adaptation for another 24 hours. Cross sections were probed with an anti-TRPL antibody (green, left column) and Alexa Fluor 546-coupled phalloidin (red, middle column). Merged panels are shown in the right column. Lower row: Localization of phosphorylation deficient TRPL8x-eGFP protein on cross sections through ommatidia derived from flies expressing the TRPL8x-eGFP construct under the control of the Rh1 promoter in a trpl 302 mutant background. Flies were aged for 3–5 days in darkness and, illuminated for 16 hours with orange light and again dark-adapted for another 24 hours. TRPL8x-eGFP was visualized by its GFP fluorescence (green, left column). Rhabdomeres were stained with Alexa Fluor 546-coupled phalloidin (red, middle column). Merged panels are shown in the right column. TRPL8x-eGFP fluorescence in the cell body appears in distinct spots (arrowheads). Scale bar: 5 μm. (TIF) [file pgen.1005578.s002.tif]

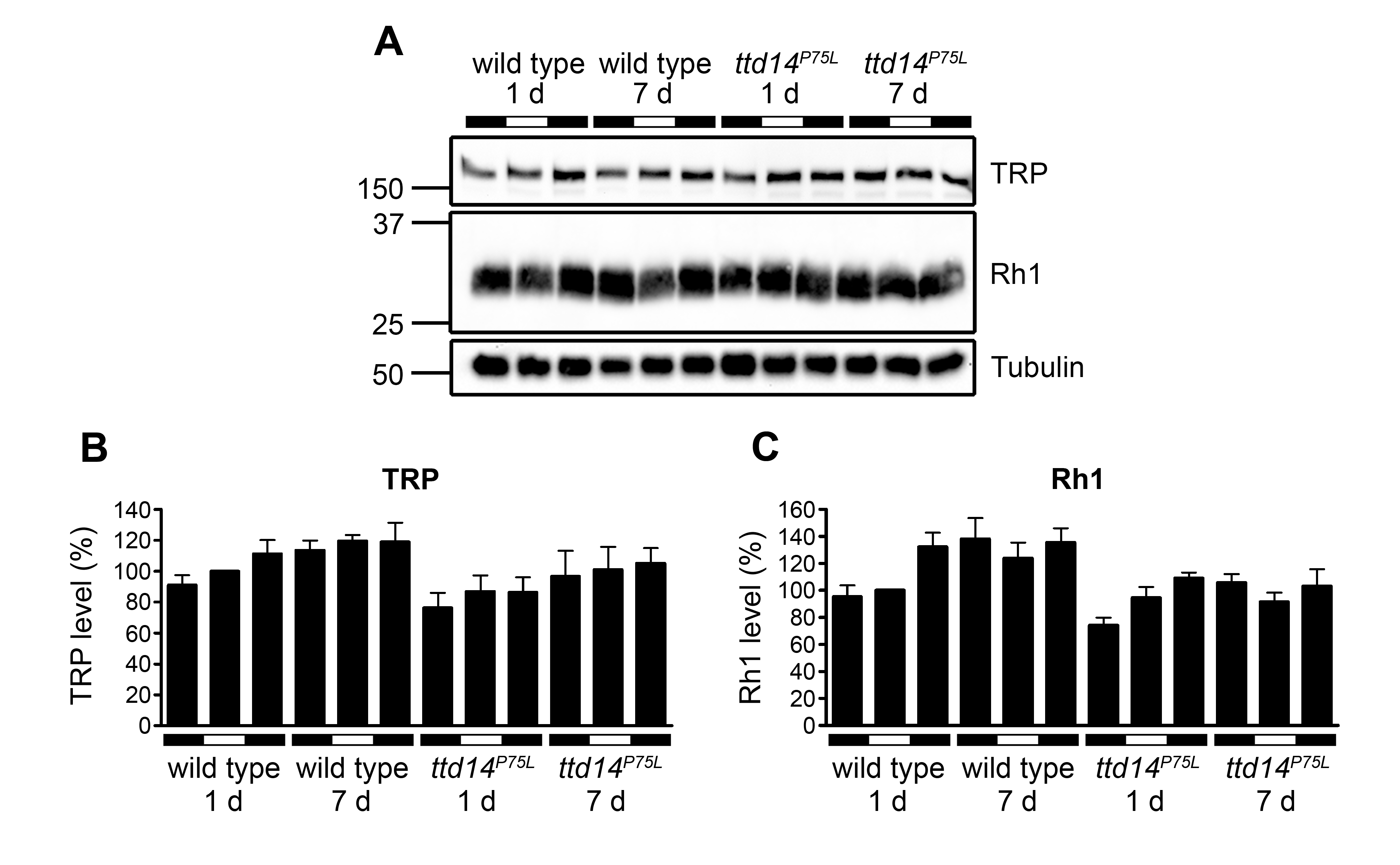

Supplement: S3 Fig — (A) Immunoblot analysis of TRP, Rh1 and Tubulin from wild type heads or from heads with ttd14 P75L mutant eye clones (equivalent of 3 heads per lane) (same blot as in Fig 4D). Freshly eclosed flies (1 day) or flies kept in the dark for 7 days were analyzed immediately (first black bars) or subjected to orange light illumination for 16 hours (white bars) followed by 24 hours of darkness (second black bars). The blots were probed with α-TRP, α-Rh1 and α-Tubulin antibodies as indicated. The size of molecular weight markers in kilo Dalton is shown at the left. (B,C) Quantification of the TRP (B) and Rh1 (C) levels normalized to Tubulin. The TRP and Rh1 levels of 1 day old flies illuminated for 16 hours (second column) was set to 100% each. Error bars show SEM (n = 5). No significant differences in the amount of TRP and Rh1 could be detected between wild type and ttd14 P75L mutant flies. (TIF) [file pgen.1005578.s003.tif]

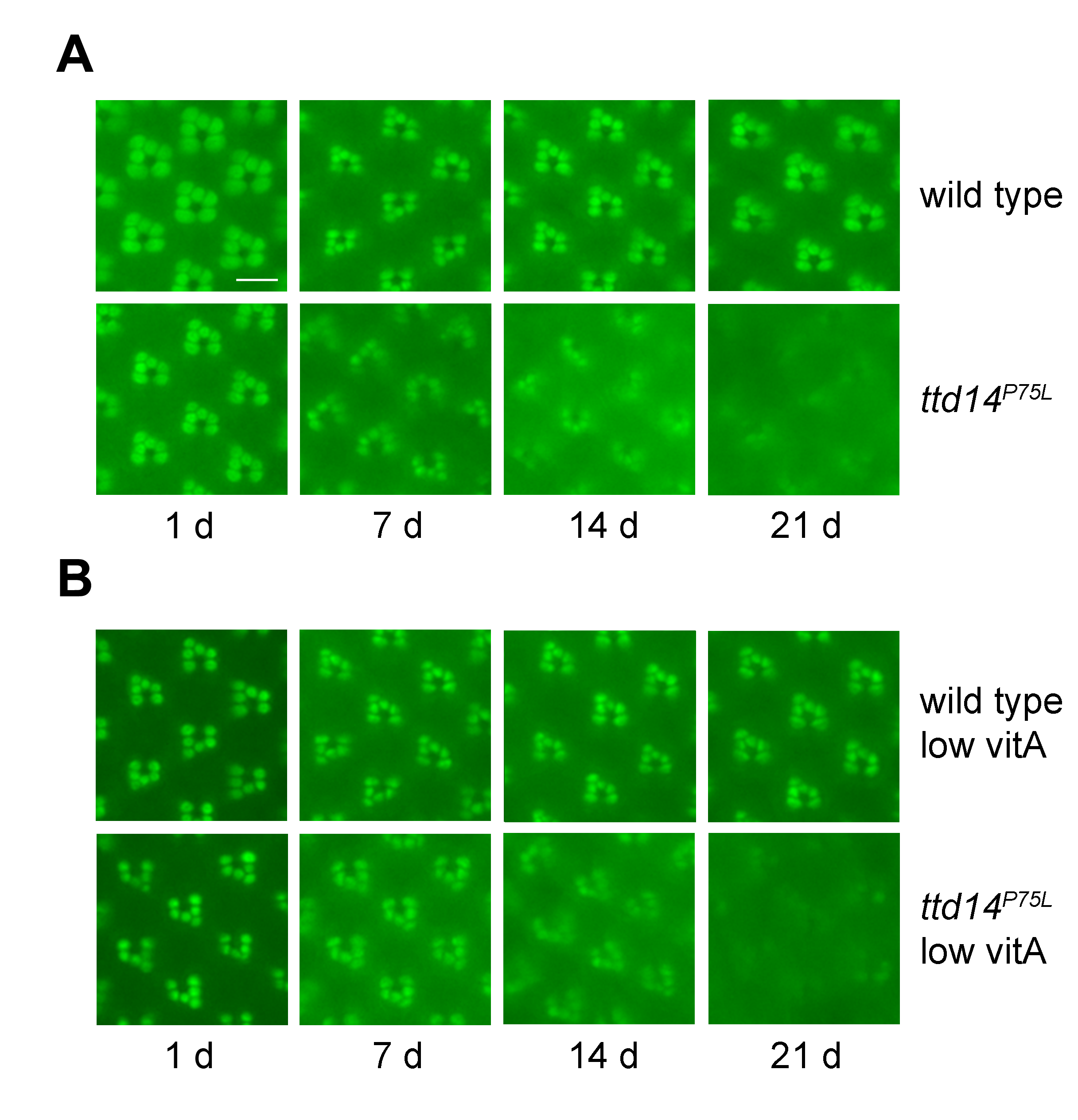

Supplement: S4 Fig — Flies were aged for the indicated number of days in a 12 hours light / 12 hours dark cycle and kept either on regular food (A) or on a vitamin A-deprived diet (low vitA) (B). Progressive loss of rhabdomeres and the regular rhabdomeral structure was observed in the ttd14 P75L mutant eye clones both on regular and vitamin-deprived food, but not in wild type eyes. Scale bar: 10 μm. (TIF) [file pgen.1005578.s004.tif]
