# Supplementary material for: The GTP- and Phospholipid-Binding Protein TTD14 Regulates Trafficking of the TRPL Ion Channel in Drosophila Photoreceptor Cells
Source: PLoS Genet. 2015 Oct 28;11(10):e1005578. doi: 10.1371/journal.pgen.1005578 (PMC4624897; doi:10.1371/journal.pgen.1005578)
Supplement: S1 Table — (DOCX) [file pgen.1005578.s005.docx]

**Table S1: Domains in vertebrate proteins that are homologous to *Drosophila* TTD14**

| **Protein*** | **AC**^#^ **number** | **Region homologous to TTD14** | **Homologous Region in TTD14** | **Sequence identity^†^** | **Sequence similarity^†^** | **Predicted domains (ProSite)^‡^** | **Domain location** | **Predicted domains**  **(InterPro)^§^** | **Domain location** |
| --- | --- | --- | --- | --- | --- | --- | --- | --- | --- |
| Mito-chondrial GTPase Era | cfT0NS81  hs[O75616](http://www.uniprot.org/uniprot/O75616)  mmQ9CZU4 | 108-161  108-161  108-161 | 61-122  61-122  61-122 | 37 %  35 %  35% | 53 %  52%  52% | Era-type guanine nucleotide-binding domain (P-loop GTPases) | 112-289  112-330  112-330 | P-loop containing NTPase | 96-199 &  225-288  100-260  97-260 |
| Sorting nexin-8 | drK9ISK3  **hsQ9Y5X2**  **mmQ8CFD4** | 112-176  127-191  122-186 | 254-320  254-320  254-320 | 33 %  30 %  34% | 56 %  52%  49% | Phox homologous- (PX) domain | 58-166  73-181  68-176 | Phox homologous- (PX) domain | 50-167  65-181  60-178 |

*Homologous vertebrate proteins were obtained by a SIB BLAST search (http://web.expasy.org/blast) using the BLOSUM62 matrix and Gap existence and extension penalties of 11 and 1, respectively

^#^UniProt Accession number of the vertebrate proteins. cf: Camelus ferus**,** hs: Homo sapiens, mm: Mus musculus, dr: Desmodus rotundus

^†^as given in the BLAST result file

**^‡^**domains predicted by Prosite (http://prosite.expasy.org) or ^§^InterPro (http://www.ebi.ac.uk/interpro) that overlap with the homologous region
